# Chemical and microbiological insights into two littoral Antarctic demosponge species: *Haliclona* (*Rhizoniera*) *dancoi* (Topsent 1901) and *Haliclona* (*Rhizoniera*) *scotti* (Kirkpatrick 1907)

**DOI:** 10.3389/fmicb.2024.1341641

**Published:** 2024-02-09

**Authors:** Maria Papale, Stefania Giannarelli, Maurizio Azzaro di Rosamarina, Lisa Ghezzi, Angelina Lo Giudice, Carmen Rizzo

**Affiliations:** ^1^Institute of Polar Sciences, National Research Council, Messina, Italy; ^2^Department of Chemical and Industrial Chemistry, University of Pisa, Pisa, Italy; ^3^Department of Earth Sciences, University of Pisa, Pisa, Italy; ^4^Stazione Zoologica Anton Dohrn, Sicily Marine Centre, Messina, Italy

**Keywords:** *Haliclona* spp., persistent organic pollutants, trace metals, prokaryotic communities, Antarctic Porifera

## Abstract

**Introduction:**

Antarctic Porifera have gained increasing interest as hosts of diversified associated microbial communities that could provide interesting insights on the holobiome system and its relation with environmental parameters.

**Methods:**

The Antarctic demosponge species *Haliclona dancoi* and *Haliclona scotti* were targeted for the determination of persistent organic pollutant (i. e., polychlorobiphenyls, PCBs, and polycyclic aromatic hydrocarbons, PAHs) and trace metal concentrations, along with the characterization of the associated prokaryotic communities by the 16S rRNA next generation sequencing, to evaluate possible relationships between pollutant accumulation (e.g., as a stress factor) and prokaryotic community composition in Antarctic sponges. To the best of our knowledge, this approach has been never applied before.

**Results:**

Notably, both chemical and microbiological data on *H. scotti* (a quite rare species in the Ross Sea) are here reported for the first time, as well as the determination of PAHs in Antarctic Porifera. Both sponge species generally contained higher amounts of pollutants than the surrounding sediment and seawater, thus demonstrating their accumulation capability. The structure of the associated prokaryotic communities, even if differing at order and genus levels between the two sponge species, was dominated by *Proteobacteria* and *Bacteroidota* (with *Archaea* abundances that were negligible) and appeared in sharp contrast to communities inhabiting the bulk environment.

**Discussions:**

Results suggested that some bacterial groups associated with *H. dancoi* and *H. scotti* were significantly (positively or negatively) correlated to the occurrence of certain contaminants.

## Introduction

The long temporal and biogeographical isolation of the Southern Ocean from other seas contributes to the strong diversity of zoobenthic marine communities in this region, characterized by a high endemism rate (McClintock et al., 2005; Kersken et al., 2016; Costa et al., 2023). Porifera, covering about 55% of the substrate in Antarctic shelves, emerge as predominant in both abundance and biomass, with ~390 described species (44% of the total species are endemic) (Downey et al., 2012). They play pivotal roles in benthic community dynamics, by providing heterogeneous three-dimensional architectures and spatial complexity, along with being involved in benthic-pelagic coupling (Schiaparelli et al., 2003; McClintock et al., 2005; Kersken et al., 2016).

Recently, Antarctic Porifera have generated increased interest from a microbiological perspective, highlighting their extraordinary plasticity as hosts of a diverse range of associated microorganisms with their surface and/or within their tissues (e.g., bacteria, diatoms, dinoflagellates) (e.g., Regoli et al., 2004; Rodríguez-Marconi et al., 2015; Cárdenas et al., 2018; Papale et al., 2020; Cristi et al., 2022). In a pioneering study using a molecular approach to describe the associated bacterial communities, Webster et al. (2004) first demonstrated that a significant portion of the retrieved diversity was sponge-specific. Subsequent investigations, including the characterization of bacterial isolates (e.g., Mangano et al., 2009, 2014; Papaleo et al., 2012; Caruso et al., 2018; Savoca et al., 2019), have seldom applied high-throughput molecular approaches to describe the microbiome associated with Antarctic sponges (e.g., Rodríguez-Marconi et al., 2015; Cárdenas et al., 2018, 2019; Sacristán-Soriano et al., 2020; Ruocco et al., 2021; Cristi et al., 2022; Happel et al., 2022). Overall, these studies have confirmed a high degree of host specificity, reveling differences from tropical and temperate sponges, such as the absence of *Cyanobacteria* and *Poribacteria*, and the dominance of *Proteobacteria* (mostly *Alpha*- and *Gammaproteobacteria*). Moreover, the microbiomes (including Bacteria, Archaea and Eukarya) of Antarctic marine sponges were found to be distinct from those of their temperate and tropical counterparts (Sacristán-Soriano et al., 2020). Functional insights highlighted that Antarctic sponge-associated prokaryotes play important roles in processes such as nutrient cycling, establishment of symbiosis, biosynthesis of secondary metabolites (including antibiotics), quorum sensing, alongside their involvement in the biodegradation of aromatic compounds (Steinert et al., 2019; Moreno-Pino et al., 2020; Papale et al., 2020; Cristi et al., 2022).

Due to their filter-feeding habit, sponges not only recruit symbiotic microorganisms but also retain bacteria, phytoplankton, and organic matter for their sustenance, while potentially accumulating in their mesohyl tissues pollutants associated with water-borne particles. This makes them potential bioindicators of pollution (Perez et al., 2003; Stabili et al., 2008). Despite its remoteness, Antarctica is not exempt from anthropogenic contamination. In fact, it is even increasingly affected by human activities at local (including tourism and research) and global scales (e.g., due to cold condensation, global fractionation, and long-range atmospheric transportation) which may prejudice its environmental, scientific and historic values (Caruso et al., 2022a). Evidence of pollutants, such as persistent organic pollutants (POPs; Vetter and Janussen, 2005; Pala et al., 2023), heavy metals (Bargagli et al., 1996; de Moreno et al., 1997; Negri et al., 2006; Illuminati et al., 2016) and microplastics (e.g., synthetic microfibers; Corti et al., 2023), were seldom reported in Antarctic sponges. The fate of pollutants may be strictly linked to microorganisms (possessing genetic and biochemical capacities for the remediation of pollutants) which constitute the first step in the transfer of toxic compounds to higher trophic levels. It is expected that microbes associated with sponges may have to cope with the presence of contaminants in the host tissues. However, to the best of our knowledge, contamination level and prokaryotic community composition in Antarctic sponges have been never treated together in the same study.

Sponges of the family Chalinidae (a.k.a. Haliclonidae; De Weerdt, 2002) are quite common in the marine environments, even in Antarctica (Barthel and Gutt, 1992). They have been proven to be a prolific source of secondary metabolites (mainly alkaloids) with several bioactivities, including antifungal, antibacterial, antiviral, anti-inflammatory, antimalarial, cytotoxic, and hemolytic activities (Avila et al., 2022; Caruso et al., 2022b). In particular, within Chalinidae, Antarctic *Haliclona* spp. have been rarely studied for prokaryotic diversity (e.g., Savoca et al., 2019; Papale et al., 2020; Ruocco et al., 2021), including the whole community and the cultivable fraction, along with the contamination level (Illuminati et al., 2016; Pala et al., 2023).

In this context, this study aimed at assessing the prokaryotic community composition, alongside the bioaccumulation of pollutants (including polychlorinated biphenyls, polycyclic aromatic hydrocarbons, and trace elements), in two Antarctic sponge species, namely *Haliclona* (*Rhizoniera*) *dancoi* (Topsent 1901) and *H*. (*Rhizoniera*) *scotti* (Kirkpatrick 1907) collected in the Thetys Bay (Terra Nova Bay, Ross Sea). Notably, *H. scotti* is a quite rare species in the Ross Sea; recently, it was found in the Thetys Bay after 114 years from its original description in 1907, and then re-described (Costa et al., 2023). The main objectives of the present study were: (1) to individuate potential differences between the two sponge species in terms of associated prokaryotes and bioaccumulation of targeted pollutants, also in relation to data obtained for seawater and sediment; (2) to preliminarily evaluate the possible relationship between pollutant accumulation (e.g., as an anthropogenic stress factor) and prokaryotic community composition in both sponge species.

## Materials and methods

### Sampling area

Thetys Bay, located nearby the Italian Mario Zucchelli Station (MZS), is a small inlet extending 3 km from its inner to outer boundaries. It is connected to the open polynya waters of the Terra Nova Bay (Ross Sea). The sea bottom (10–50 m depth) features a mix of partial rocky and muddy cover, where the benthic vegetation is scarce (Calizza et al., 2018). The sea- ice dynamics exhibit a marked seasonality, with the absence of ice during the austral summer and an ice cover that generally last until December. This seasonal pattern strongly affects primary productivity and the development of phytoplankton blooms and, in turn, the food supply to benthic communities, with repercussions on their distribution (Pusceddu et al., 1999).

### Collection and preliminary treatment of samples

**Figure 1:**
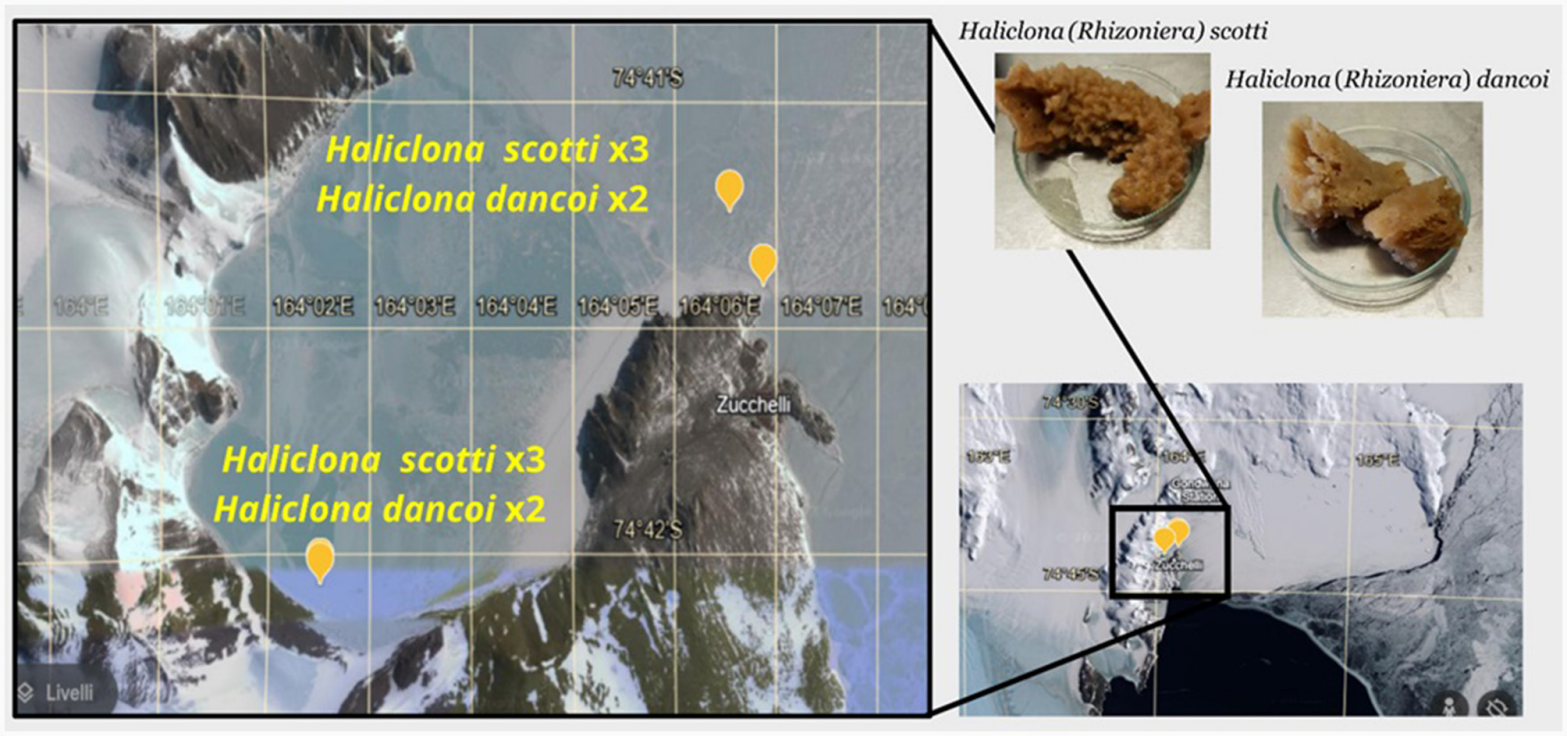
Maps of the study area showing the sampling sites and sponge specimens collected.

Samples of the Antarctic sponges *Haliclona dancoi* and *H. scotti* were aseptically collected by scuba divers from the Thetys Bay in 2018 (inner bay) and 2019 (outer bay) during the XXXIII and XXXIV Italian Expeditions to Antarctica (*n* = 9), respectively (Table 1; Figure 1). Fragments of sponge individuals were packed underwater in sterile plastic bags and stored at +4°C for the transportation to the laboratory for preliminary processing (within 2 h after sampling). Sponge surfaces were rinsed at least three times with filter-sterilized seawater and dissected (Mangano et al., 2009; Steinert et al., 2019). Fragments of each specimen were then preserved at −20°C for DNA extraction and chemical analyses (in aluminum foils), and in 70% ethanol for taxonomic classification (previously reported by Costa et al., 2023). The collection of sponges was previously authorized by the Programma Nazionale di Ricerche in Antartide (PNRA), conformably to the Antarctic Treaty legislation and the SCAR Code of Conduct for the Use of Animals for Scientific Purposes. A fragment of each sponge individual was deposited at the Italian National Antarctic Museum (MNA, Section of Genoa, Italy) under the MNA voucher codes listed in Table 1. Seawater and sediment samples were contextually collected in a 10 cm radius from the sponge individuals. For the subsequent microbiological analyses, seawater samples (between 1.5 and 2.0 L) were filtered on polycarbonate membranes (diameter 47 mm; 0.22 μm) and stored at −20°C until processing, while sediment samples were directly stored at −20°C in sterile containers. For the extraction of persistent organic pollutants, sediment samples were collected with an aluminum scoop, placed in aluminum foil (previously decontaminated by washing with acetone and subsequently with hexane), and then stored at −20°C until analysis. For trace metal analysis, sediments were directly collected by using plastic containers, and then stored at −20°C until analysis. Seawater samples were collected in duplicate using 25 L stainless steel bottles. The bottles were then taken to the laboratories of MZS where the extraction took place within 1–2 days from the moment of sampling (*see below*), with the aim of minimizing any changes in the analytes for effect of prolonged storage.

**Table 1:** Biotic and abiotic samples analyzed in this study.

**Sponge species/abiotic matrix**	**Antarctic Expedition/Year**	**Coordinates**	**Sample ID**	**MNA code**
*Haliclona dancoi*	XXXIII/2018	74°42′04″S; 164°02′31″E	B1	MNA-11767
			B3	MNA-11769
	XXXIV/2019	74°41,252′S; 164°06,548′E	1Sp2a	MNA-11747
*Haliclona scotti*	XXXIII/2018	74°42′04″S; 164°02′31″E	C1	MNA-11770
			C2	MNA-11771
			C3	MNA-11772
	XXXIV/2019	74°41,252′S; 164°06,548′E	1Sp1a	MNA-11744
			1Sp1b	MNA-11745
			1Sp1c	MNA-11746
Sediment	XXXIII/2018	74°42′04″S; 164°02′31″E	SED18a	—
	XXXIV/2019	74°41,252′S; 164°06,548′E	SED19a	—
		74°41,416′S; 164°06,880′E	SED19b	—
Seawater	XXXIII/2018	74°42′04″S; 164°02′31″E	WAT18a	—
	XXXIV/2019	74°41,252′S; 164°06,548′E	WAT19a	—
		74°41,416′S; 164°06,880′E	WAT19b	—

### Persistent organic pollutant analysis

**Figure 2:**
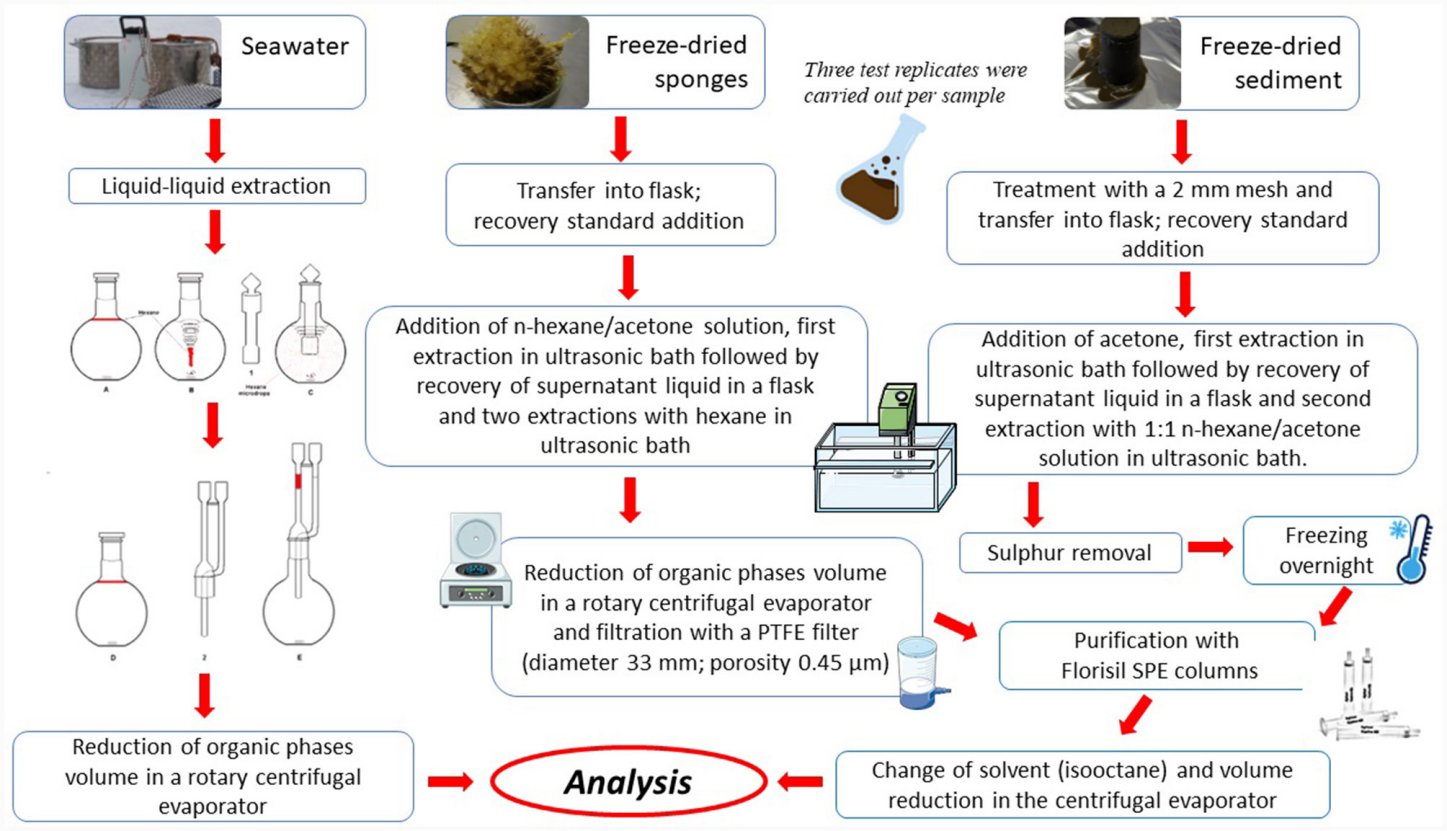
Scheme of the extraction procedure applied to seawater, sponge and sediment samples prior to be analyzed.

Among POPs, polychlorobiphenyls (PCBs) and polycyclic aromatic hydrocarbons (PAHs) were determined in sponge, water and sediment samples. However, due to logistic constrains, seawater samples were not collected in 2018 for POP extraction. The simplified workflow of the extraction procedure is reported in Figure 2.

#### Chemicals

Isooctane, n-hexane and acetone were Pestanal from Riedel-De Haen; double distilled water HPLC quality was from Fluka Analytical, Sigma Aldrich; the sodium sulfate was from Carlo Erba Reagents; TBA sulphite and 2-propanol were from Sigma Aldrich. The internal standard, used to determine the recovery of the sample treatment from the extraction to analysis, was obtained from L429-IS (Wellington Laboratories, deuterated PAHs) and ^13^C labeled PCB mixture (EC-4058, Cambridge Isotope Laboratories). The injection standard to correct instrumental variability was obtained using the following certified solutions: Recovery Standard Stock Solution and Alternate Standard Stock Solution (L429-RS and L429-AS, respectively, Wellington Laboratories) with deuterated PAHs and MBP-MXF solution (Wellington Laboratories, ^13^C-PCBs). The following solutions were used as standard solutions for calibrations: certified Native Stock IPA Solution (L429-PAR, Wellington Laboratories for PAHs); Native PCB Precision and Recovery Solution (EC-9605-PAR, Wellington Laboratories); PCB 15, PCB 31, PCB 99, PCB 110, PCB 113, PCB 132, and PCB 158 by Labor. Dr. Ehrenstorfer, and PCB 89, PCB 95, PCB 149, PCB 151, and PCB 156 from Cambridge Isotope Laboratories; Custom Pesticide Mix from Smart Solutions for pesticides. The standard solutions for the calibration curves were prepared by dilution starting from a stock solution mixture of PAHs and PCBs in isooctane, obtained by subsequent dilution of the latter. All solutions were stored in a refrigerator at 4°C. All reagents and chemicals were used without further purification.

#### Extraction of sponge samples and extract purification

Sponge samples were freeze-dried (dry sponge weight was on average 16% of total weight) and homogenized with the help of a mortar. A known volume (10 μl) of method standard (MET) was added to each sample (3 g) prior to extraction. A mixture (30 ml) of n-hexane/acetone 1:1 was added to perform a solid-liquid extraction using an ultrasonic bath for 40 min at 45°C. Once back to room temperature, the supernatant was recovered and two further extractions were carried out. The organic phases recovered from the three extractions were combined and reduced to a volume of 5 ml, by using a rotary centrifugal evaporator. After centrifugation (5,000 rpm for 30 min), the recovered solution was concentrated to ~3 ml. The extracts were then purified on SPE cartridges after the construction of an elution curve. For all porifera samples, 5 ml of hexane were used to elute the sample through the SPE column. A procedural blank was also performed, using all the reagents and solvents foreseen by the procedure, in the absence of the sponge sample, following the same procedures described above. The results of the blank determination were used to correct sample measurements or to detect errors due to interference from contaminants present in the reagents. Three replicate tests were carried out *per* sample.

#### Extraction of sediment samples and extract purification

The total humidity of the sediment samples was determined by oven drying: the percentage of residual water present in the samples varied around an average of 23%.

Sediment samples were left to thaw overnight in an ISO 5 clean lab, leaving them in the original aluminum containers. Then sediments were treated with a 2 mm mesh sieve and a known volume (10 μl) of MET was added to each sample (30 g) before extraction. After the addition of acetone (50 ml), a solid-liquid extraction was performed using an ultrasonic bath for 30 min at 60°C. Acetone was used to facilitate the subsequent extraction action of hexane, increasing the wettability of the sediments. Once returned to room temperature, the supernatant was recovered and a 1:1 n-hexane/acetone solution (40 ml) was added to each sediment sample, carrying out a second extraction in the ultrasonic bath for 30 min at 60°C. The two organic phases were combined and subsequently reduced to a volume of 10 ml, by using a rotary centrifugal evaporator. Samples were filtered with a PTFE filter (diameter 33 mm; porosity 0.45 μm) to eliminate suspended particles, after conditioning the filter with hexane, and subsequently reduced to ~2 ml.

To remove sulfur, we use an efficient, rapid, non-toxic, and non-destructive way for analytes of interest (Jersen et al., 1977); briefly, a solution was prepared by adding 3.39 g of tetrabutylammonium sulphite to 100 ml of double distilled water; subsequently three washes were performed with 20 ml of hexane each; the solution was then saturated with 25 g of sodium sulphite. The solution was placed in a dark glass bottle and stored at room temperature.

To each extract (2 ml), 1 ml of 2-propanol and 1 ml of TBA solution were added, following vortexing for 1 min. Sodium sulphite was added until saturation. Double-distilled water (5 ml) was added, followed by a for 1 min stirring. The solution was left to rest for about 10 min to allow the phases (i.e., water and organic) to be sorted. After freezing overnight, the organic phase was recovered and the extracts were purified with Si SPE columns, activated with ~2 ml of hexane. After sample recovery, 2 ml of isooctane were added and the samples were reduced, by using a centrifugal evaporator, to ~1 ml in volume. Checked the volume for weigh, a spike of standard injection (INJ), equal to one hundred of the volume, was added to each sample, using a graduated micro syringe. A procedural blank was also performed, using all the reagents and solvents foreseen by the procedure, in the absence of the sediment sample, following the same procedures described above. Three test replicates *per* sample were carried out.

#### Extraction of seawater samples and extract purification

Before extraction, a known amount of the “method-standard” solution was added to the samples. The samples were immediately extracted twice with 20 ml of n-hexane using a custom-made extraction system (Zoccolillo et al., 2004). The two aliquots of organic phase were recovered and combined, and the volume of the extracted water was measured accurately. The organic phases were then stored in glass containers at −20°C until their arrival in Italy. Once in the analytical laboratory, the extracts were treated with anhydrous Na_2_SO_4_ immediately before the analysis. After recovering the solution, a solvent exchange was performed by adding 1 ml of isooctane and reducing the volume of the sample to about 1 ml in a centrifuge vacuum evaporator. Finally, a known amount of the “injection-standard” solution was added to the sample. A blank procedure was also carried out using pure water as a sample.

#### Gas-chromatography/mass-spectrometry analyses

Standard solution analysis was carried out to optimize instrumental parameters in TIC (Total Ion Current) mode. Then, an Multiple Reaction Monitoring (MRM) program was created: two different precursor ion—product ion transitions were chosen for each analyte, the most intense was selected as “quantifier,” while the other as “qualifier.” Retention times and selected transitions are given in the Supplementary Table S1.

The instrument used was an Agilent GC 7890B coupled with a MS 7010 triple quadrupole mass spectrometer, equipped with an Agilent ALS autosampler and an MMI (Multi Mode Injector) injection port, used in the solvent vent mode. The column used was an HP-5MS UI (95% dimethyl – 5% phenylpolysiloxane, 30 m × 0.250 mm, film thickness 0.25 μm). The mobile phase was helium, with a flow of 1.2 ml/min, a pressure of 11,052 psi. For the cell of collision, helium was used with a flow of 4.0 ml/min, while nitrogen was used with a flow of 1.5 ml/min with a pressure of 10 psi. The injector was at an initial temperature of 85°C with the split valve open for 0.53 min for solvent evaporation. Then the split valve was closed, and the temperature was increased to 300°C at a rate of 600°C/min for 10 min. The oven temperature for the analysis was set as follows: 70°C for 3 min, 50°C/min up to 150°C, isotherm 2 min, 5°C/min up to 310°C. All analyzes were performed by injecting 2 μl of sample.

The data system contains all the software required for calibration, GC/MS-MS spectra collection and data processing for qualitative and quantitative analysis. Several field blank samples were prepared in the clean laboratory at the Italian base in Antarctica with MilliQ-grade pre-extracted water and analyzed with the same MRM procedure used for water, porifera and sediment samples. The limit of detection (LOD) and the limit of quantification (LOQ) were calculated for each compound as three times and ten times, respectively, the standard deviation of the blank (calculated on seven replicate blanks). The range of LOD and LOQ resulted 0.0001–0.001 and 0.0004–0.006, and 0.0001–0.04 and 0.0003–0.1 ng/L for PCBs and PAHs, respectively. The analytes were grouped according to the expected concentration range and the corresponding calibration curves were obtained based on the following eight concentration levels:

- ACY, ACE, FLU, PHE, ANT, F, and Py at 0.1, 0.2, 0.5, 1.0, 2.0, 5.0, 10.0, 20.0 ng/L;
- BaA, C, BbF, BkF, BaP, IP, and BP at 0.01, 0.02, 0.05, 0.1, 0.2, 0.5, 1.0, 2.0 ng/L;
- All PCBs at 0.001, 0.002, 0.005, 0.01, 0.02, 0.05, 0.1, 0.2 ng/L.

All the calibration curves resulted linear in the observed concentration range, with a typically value of *r*^2^ of 0.999, always better than 0.997.

### Trace metal analysis

Trace elements were determined in sponges and sediment samples collected in 2018 and 2019. The total mercury (Hg) content was determined by a Milestone Dma-80 Direct Mercury Analyser following the US EPA Method 7473. All analyses were carried out in triplicate. Blank were run every 10 samples and to ensure the quality of the results, the certified standards ERM-CC018 (Contaminated Sandy Soil) and MESS-3 (Marine Sediment) were used as reference materials. Relative standard deviation (RSD) and accuracy evaluated by five replicate analyses were within 5%.

Sediment (500 mg) and sponge (100 mg) samples were digested with inverse aqua regia (6 ml 65% HNO_3_ + 2 ml 37% HCl, Suprapur grade) by using a Milestone Ethos Easy microwave platform. After acid digestion, the solutions were brought up to 50 ml with Milli-Q water and appropriately diluted before analysis. The concentration of a set of trace elements (Li, Be, V, Cr, Mn, Fe, Co, Ni, Cu, Zn, As, Sr, Mo, Ag, Cd, Sn, Sb, Ba, Tl, Pb, Th, and U) was determined by ICP-MS using a Perkin Elmer NexION 300X. The analytical uncertainty was evaluated by replicate analysis (*n* = 10) of the reference material Nist 2711a and ERM-CC018. In general, the accuracy was better than 10%. Precision values, as relative standard deviation, were better than 5% for Li, Be, Mn, Ni, Ag, Sn, Cd, Tl, Pb, Fe, and As, and within 10% for Co, Cu, Zn, Sr, Sb, Ba, Th, U, V, and Cr.

### Prokaryotic community diversity and composition

#### DNA extraction

Total DNA was extracted from sponge homogenates, sediment and filter membranes (for seawater) by using the Power Soil DNA extraction kit (MoBio Laboratories, Carlsbad, CA, USA) according to the manufacturer's instructions. The concentrations and purity of extracted DNA were checked by using a NanoDrop ND-1000 UV-vis spectrophotometer (NanoDrop Technologies, Wilmington, DE, USA). The DNA extracted from each specimen was sequenced in triplicates.

#### Amplification and sequencing of 16S rRNA genes

Briefly the sequencing reaction was set up as follows using microbial DNA 2.5 μl (5 ng/μl concentration), forward and reverse primer 5 μl at a concentration of 1 μM and KAPA HiFi Hot Start Ready Mix (Roche Sequencing Solution, Milan, Italy) 12.5 μl. Polymerase chain reaction (PCR) was performed by the thermocycler Applied Biosystems 9700, following the program: 3 min at 95°C, followed by 25 cycles of 95°C for 30 s, 55°C for 30 s, and 72°C for 30 s, with a final extension at 72°C for 5 min. The Agilent 2100 bioanalyzer with a DNA 1000 chip was used to verify PCR size. AMPure XP beads were used to purify the amplicon product from the free primers and primer dimer species. The DNA concentration of each PCR product was determined using a Qubit^®^ 2.0 Green doublestranded DNA assay. Depending on the coverage needs, all libraries could be pooled for one run. The amplicons from each reaction mixture were pooled in equimolar ratios based on their concentrations. Bacterial 16S rDNA region V3-V4 was amplified using universal primers 341F 50-CCTACGGGNGGCWGCAG-30 and 805 R 50-GGACTACHVGGGTATCTAATCC-30 (Kozich et al., 2013), incorporated by a Nextera XT Index kit (Illumina, San Diego, CA, USA). Libraries were normalized based on fragment length and dsDNA molarity. The normalized samples were combined and processed in four sessions using a MiSeq platform (Illumina) and MiSeq reagent kit v3-600 for 2 × 300 paired-end sequencing at the IGA Technology Services Srl (Udine, Italy).

#### Post-run analysis

Raw sequences were quality-checked using the FastQC tool (Brown et al., 2017). All subsequent analysis steps (quality filtered, trimmed, de-noised, merged, cleaning, and affiliation) were performed using the R package DADA2 (Weißbecker et al., 2020) to infer amplicon sequence variants (ASVs), i.e., biologically relevant variants rather than an arbitrarily clustered group of similar sequences. Particularly quality and cleaning steps were as follows: minimum length between 150 and 140 bp, no reads with *N* base were maintained in the analysis, and all sequences were trimmed at the ends after quality control, trimLeft = 17, trimRight = 15. During the analysis, filters for reducing replicate, length, and chimera errors were also applied. Bacterial taxonomy annotation was performed using Silva database formatted for DADA2, offering an updated framework for annotating microbial taxonomy (silva_nr99_v138.1_wSpecies_train_set.fa.gz and silva_species_assignment_v138.1.fa.gz). Finally, a manual inspection was done.

#### Statistical analyses

Diversity indices, namely Shannon diversity index (*H*′) (Shannon, 1948), Simpson (Simpson, 1949), Chao1 (Chao and Jost, 2012), Fisher (Fisher et al., 1943) in each sample were calculated by R package phyloseq with the estimate richness_function. Principal component analysis (PCA) was performed to compare the bacterial community compositions across groups of samples and was obtained using the factoextra R package, on data from bacterial phyla. The results of relative abundances were transformed and processed by calculating the Bray—Curtis similarity. The pollutants retrieved concentrations with retrieved bacterial phyla were then used for the Spearman correlation matrix calculation and plotting (R package corrplot), by considering results statistically significant when *p* < 0.05. The entire dataset (including chemical analysis and bacterial abundance at genus level) was used to perform a PCA showing the spatial distribution of samples according to their distance, and the correlation with related factors (Pearson correlation factor >0.9).

## Results

### Determination of POPs and trace metals

The entire dataset, including standard deviations, obtained by the determination of POPs and trace metals is reported in the Supplementary Table S2.

#### Polychlorobiphenyls

**Figure 3:**
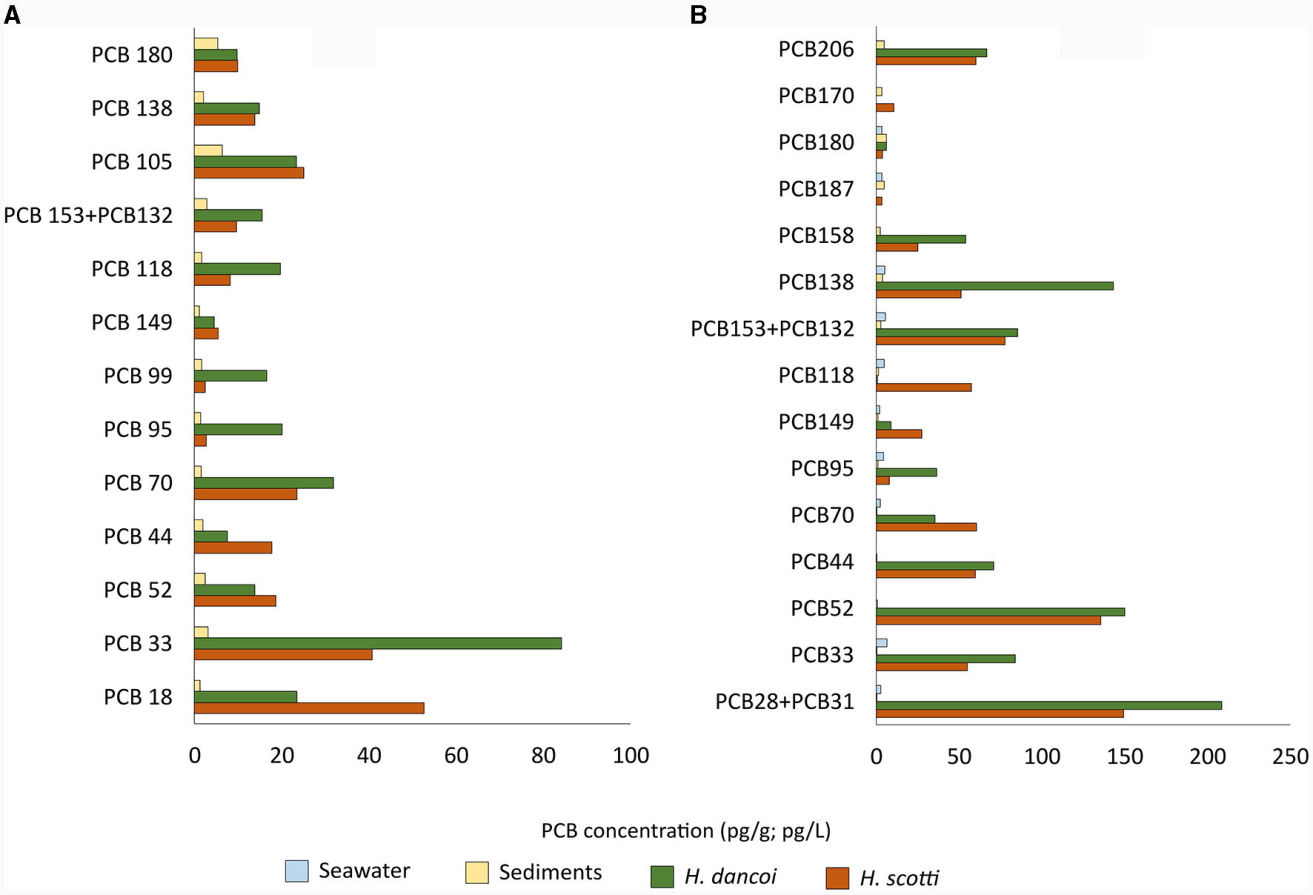
Concentrations of PCB congeners determined in seawater (expressed in pg/L), sediment and sponge samples (both expressed in pg/g) from the Thetys Bay (Terra Nova Bay, Antarctica) during austral summer **(A)** 2018 and **(B)** 2019. Only values above 1 pg/g or pg/L are shown. *Please note the different scale*.

In 2018, higher concentrations of PCBs were detected in sponge mesohyl than in sediment (50 and 1–7 pg/g, respectively). In particular, the congeners PCB18 (trichlorobiphenyls; 84.2 and 40.8 pg/g in *H. dancoi* and *H. scotti*, respectively), PCB70 (tetrachlorobiphenils; 31.9 and 23.5 pg/g in *H. dancoi* and *H. scotti*, respectively) and PCB105 (pentachlorobiphenyls; 23.3 and 25.1 pg/g in *H. dancoi* and *H. scotti*, respectively) were particularly enriched (Figure 3A).

In 2019, higher concentrations of PCB congeners were again detected in sponges than in sediment samples, with values that resulted higher than those determined in 2018. A number of congeners were particularly enriched in *H. dancoi* with respect to *H. scotti*, as follows: PCB89 + PCB101 (218.3 and 68.0 pg/g, respectively), PCB28 + PCB31 (208.6 and 149.5 pg/g, respectively), PCB52 (150.3 and 135.6 pg/g, respectively), PCB110 (192.5 pg/g in *H. dancoi*; absent in *H. scotti*). In water and sediment samples the overall PCB concentration was estimated to be about 10 pg/L and pg/g, respectively (Figure 3B).

#### Polycyclic aromatic hydrocarbons

**Figure 4:**
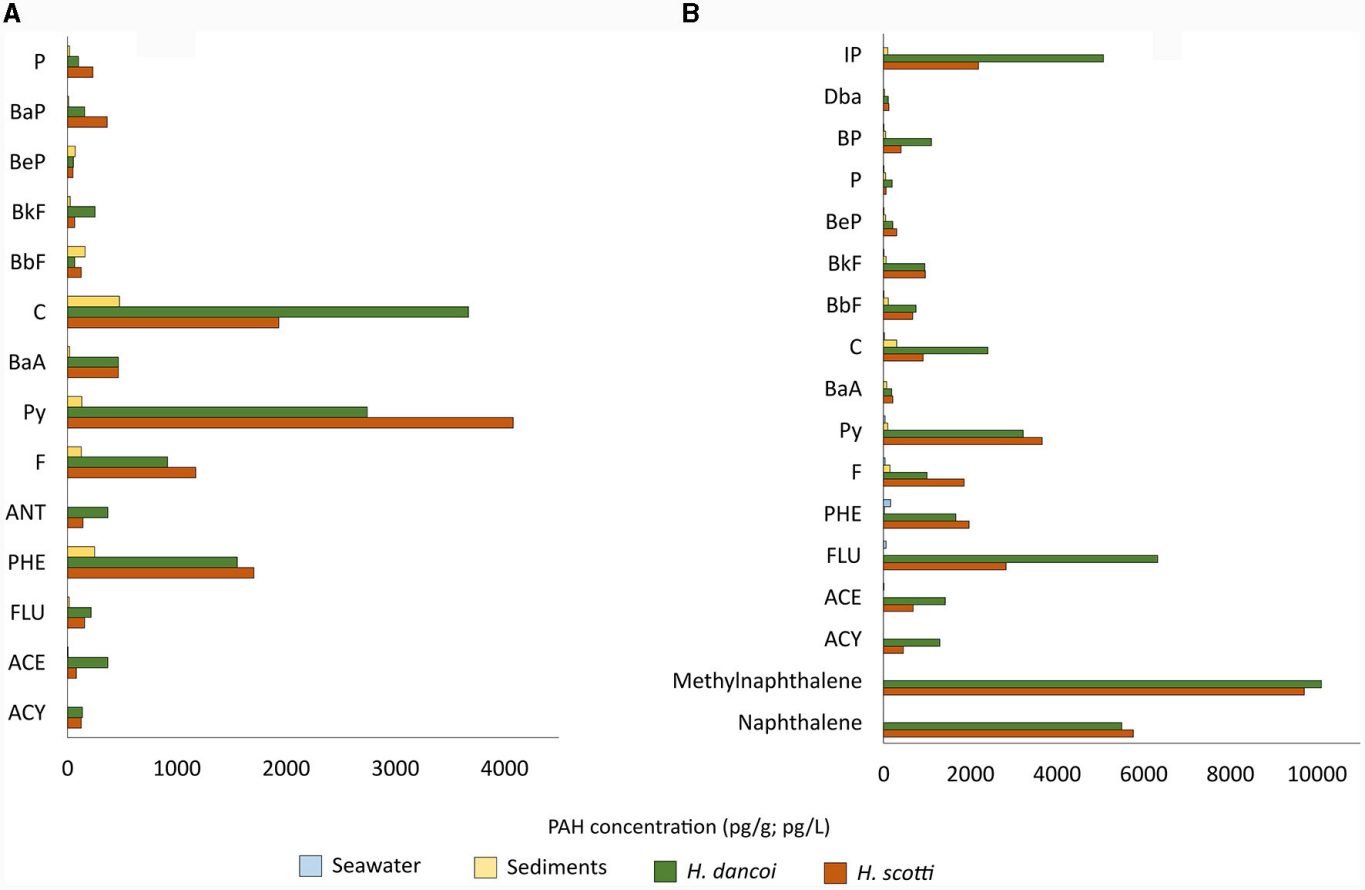
PAH concentrations retrieved in seawater (expressed in pg/L), sediment and sponge samples (both expressed in pg/g) from the Thetys Bay (Terra Nova Bay, Antarctica) during austral summer **(A)** 2018 and **(B)** 2019. Only values above 1 pg/g or pg/L are shown. *Please note the different scale*.

In 2018, as it was reported above for PCBs, PAHs were more concentrated in sponges than in sediment samples (being an order of magnitude higher). Phenanthrene (1,555.9 and 1,708.3 pg/g in *H. dancoi* and *H. scotti*, respectively), pyrene (2,744 and 4,083.6 pg/g in *H. dancoi* and *H. scotti*, respectively) and crysene (3,673.9 and 1,936.5 in *H. dancoi* and *H. scotti*, respectively) were particularly enriched in both sponge species (Figure 4A).

A similar trend in the concentration of some PAHs was observed for 2019 samples. The highest values were retrieved for naphthalene and methylnaphthalene (5,497.9 and 10,112.0 pg/g in *H. dancoi*, and 5,769.2 and 9,720.6 pg/g in *H. scotti*, respectively), followed by fluorene (6,333.2 and 2,827.5 pg/g in *H. dancoi* and *H. scotti*, respectively) and pyrene (3,229.7 and in *H. dancoi* and 3,657.6 pg/g *H. scotti*, respectively). Sediment samples showed higher concentrations of PAHs than water, with values that were about 100 pg/g (Figure 4B).

#### Trace metals

**Figure 5:**
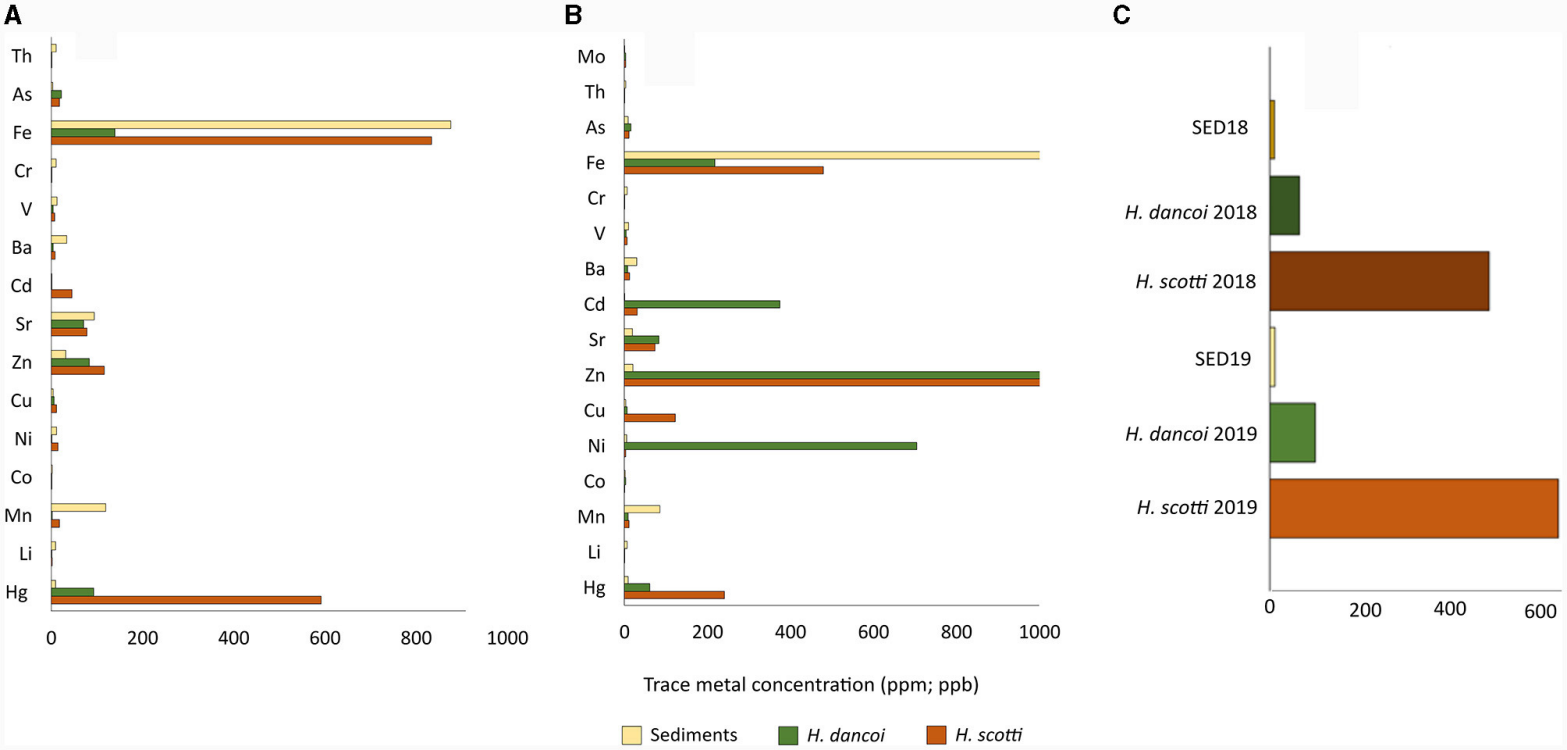
Trace metal concentrations (expressed in ppm) in sediment and sponge samples collected at Thetys Bay (Terra Nova Bay, Antarctica) during austral summer **(A)** 2018 and **(B)** 2019. Hg concentrations (ppb) in all samples are shown in **(C)**. *Please note the different scale and units*.

Samples collected in 2018 showed a high concentration of As, ranging from 8.8 mg/kg in sediments to 16.3 and 16.0 mg/kg in *H. scotti* and *H. dancoi*, respectively. A higher amount of some metals was detected in *H. dancoi* than in both *H. scotti* and sediment samples, e.g., Ni (705 mg/kg), Zn (1,854 mg/kg) and Cd (375 mg/kg). Mo was detected in equal amounts in both sponge species, with 3.7 mg/kg (Figure 5A).

In 2019, most heavy metals were found at higher concentrations in sponge tissues than in sediment samples, with few exceptions, i.e. Mn (120.3 mg/kg of sediment vs. 2.9 and 18.8 mg/kg in *H. dancoi* B and *H. scotti* C, respectively), Sr (94.4 mg/kg of sediment vs. 70.8 and 78.4 mg/kg in *H. dancoi* B and *H. scotti* C, respectively), Ba (34.4 mg/kg of sediment vs. 4.6 and 8.9 mg/kg in *H. dancoi* B and *H. scotti* C, respectively). A concentration of 22.7 and 17.9 mg/kg of As was detected in individuals of *H. dancoi* and *H. scotti*, respectively, while in sediments it was detected with a concentration of 3.3 mg/kg. The highest concentration of Cd (45.8 mg/kg) was retrieved in *H. scotti*, whilst in both *H. dancoi* and sediment samples the concentration of this metal was negligible (Figure 5B). Finally, Hg was particularly enriched in *H. scotti* (592 μg/kg) and, at a lesser extent, in *H. dancoi* (92.9 μg/kg) with respect to sediment showing a concentration of 10.3 μg/kg (Figure 5C).

### Prokaryotic community diversity and composition

**Figure 6:**
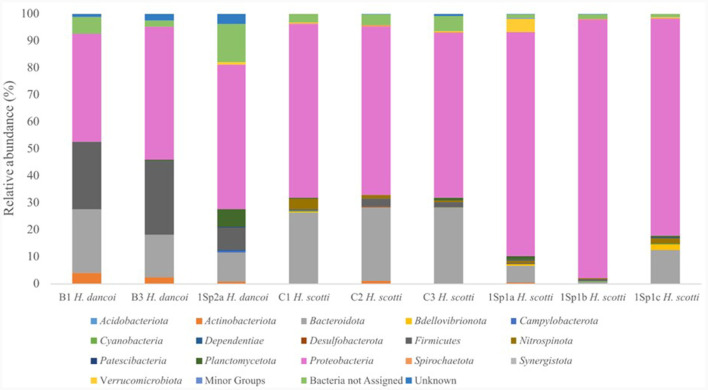
Bacterial community composition at phylum level in sponge specimens collected from the Thetys Bay.

Data on total sequence reads, quality trimming, ASV information and diversity indices obtained for samples included in this study are reported in the Supplementary Table S3. Shannon and Simpson indices showed that the diversity level was similar in all sponge samples, while the Chao1 index was lower for the samples *H. dancoi* B1 and *H. dancoi* B3. The prokaryotic communities were mainly represented by bacteria, with overall percentages of 98%−99%. The only exception was *H. dancoi* B1, collected in 2018, as bacteria predominated with the 95.7% of total microbial community and the 3.6% was represented by Archaea. Overall, the taxonomic composition of bacterial assemblages at phylum level showed the predominance of *Proteobacteria* and *Bacteroidota*, followed by *Firmicutes* and *Planctomycetota* (Figure 6). The taxonomic structure of microbial communities is detailed in the following sections. The abundance of each group is expressed as relative abundance within the total bacterial/archaeal community.

#### Haliclona dancoi

The specimens of *H. dancoi* sampled in 2018, namely B1 and B3, showed predominance of *Proteobacteria* (abundance of 40.0 and 49.2%) and *Bacteroidota* (40.0 and 49.2%), respectively. Similarly, the specimen collected in the 2019 (namely 1Sp2a) showed *Proteobacteria* and *Bacteroidota* average relative abundances of 53.5 and 10.8%, respectively. *Firmicutes* occurred at higher abundance in specimens of *H. dancoi* B1 and B2, accounting for 25.1 and 27.5%, respectively, while an average abundance of 8.2% was detected in the specimen collected in 2019. Conversely, *Planctomycetota* were more represented in *H. dancoi* collected in 2018, with a relative abundance of 6.4% (Figure 6).

**Figure 7:**
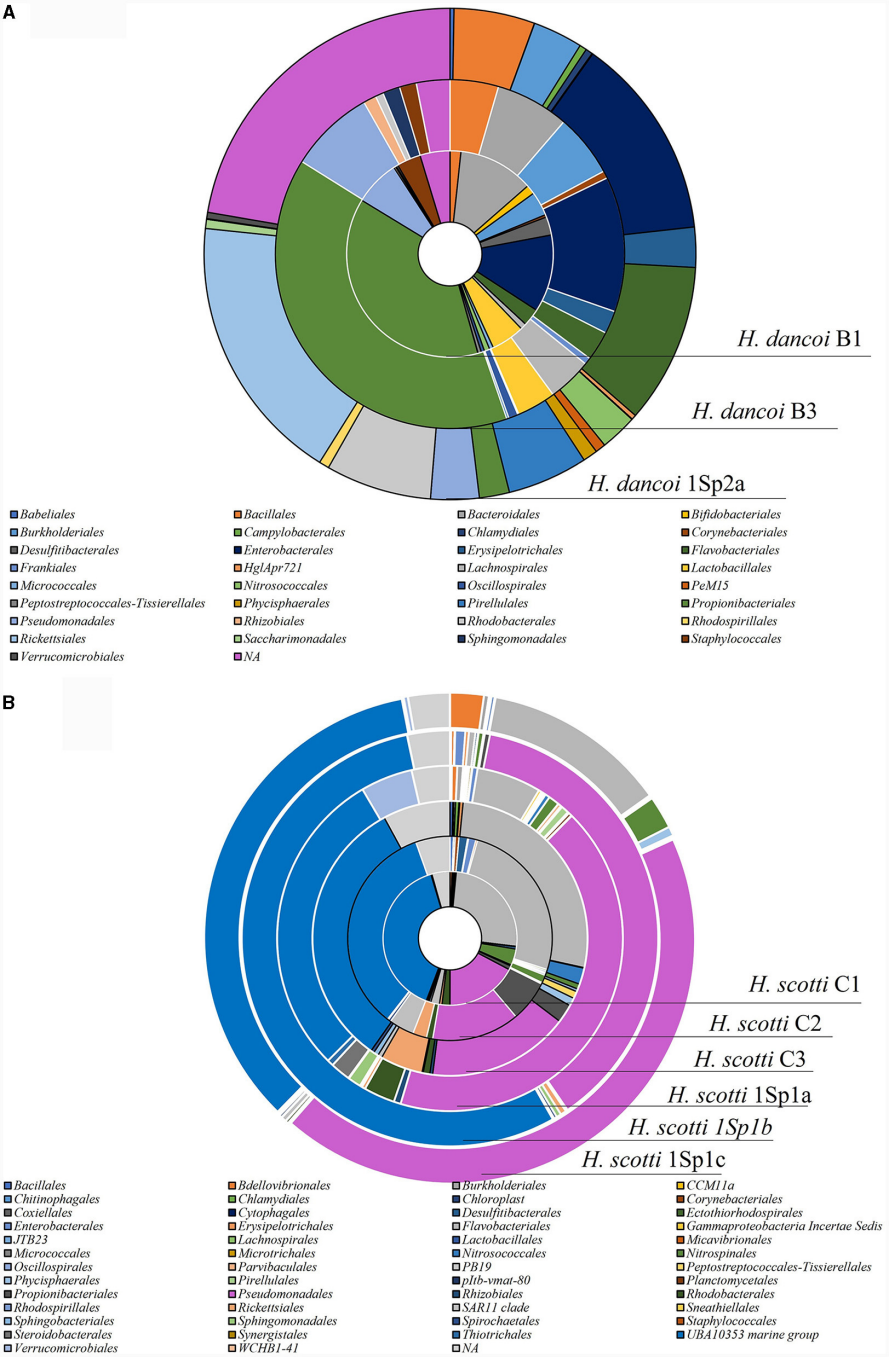
Taxonomic composition at order level of the bacterial communities associated with **(A)** *Haliclona dancoi* and **(B)** *Haliclona scotti* specimens collected from the Tethys Bay (Antarctica).

Differences in the relative abundance of some taxonomic groups were observed between 2018 and 2019 samplings at the order level (Supplementary Table S4). For instance, *Propionibacteriales* were more abundant in specimens collected in 2018 (relative abundance of 38 and 39.1%, in B1 and B3, respectively) than 2019 (2% in 1Sp2a). Similarly, *Bacteroidales* were abundant in 2018 (11.8 and 6.8% in B1 and B3, respectively), but they were absent in 1Sp2a collected in 2019. *Flabobacteriales* accounted for 10.5 and 2.7% in 1Sp2a and both *H. dancoi* specimens collected in 2018. Conversely, *Rickettsiales* and *Rhodobacterales* were quite absent in the sponges collected in 2018 but resulted abundant (17.7 and 6.9%, respectively) in 1Sp2a collected in 2019. *Burkholderiales* and *Enterobacterales* showed similar abundances in samples collected in both years (with abundances ranging from 3.3 to 5.8% and from 12.1 to 13.3% in 2018 and 2019, respectively (Figure 7A).

**Figure 8:**
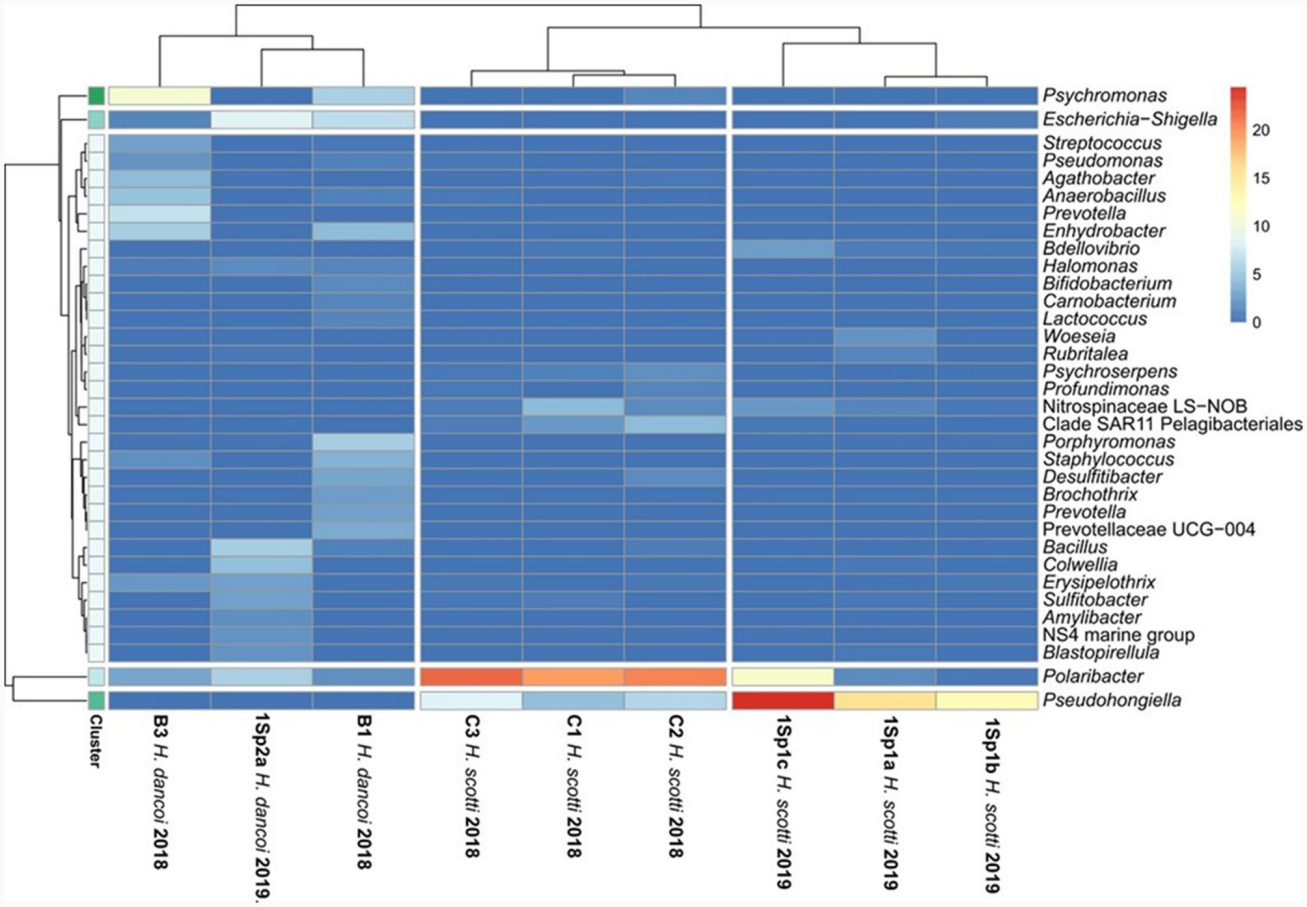
Heatmap showing the taxonomic composition of sponge-associated bacterial communities at genus level.

*Escherichia-Shigella, Psychromonas, Colwellia, Enhydrobacter* (within *Gammaproteobacteria*), *Polaribacter, Porphyromonas* (within *Bacteroidota*), *Agathobacter, Anaerobacillus, Bacillus, Staphylococcus* (within *Firmicutes*) were the most abundant genera (Figure 8).

Finally, the Archaeal community was exclusively represented by *Candidatus Nitrosopumilus* (Group 1a *Nitrososphaerota*, formerly *Thaumarchaeota*) in all *H. dancoi* specimens.

#### Haliclona scotti

*Proteobacteria* and *Bacteroidota* dominated also the bacterial communities associated with *H. scotti*. Specifically, *Proteobacteria* sequences accounted between 60.9 and 64.3% in samples collected in 2018, while they ranged from 80.5 to 95.7% in association with sponge specimens collected in 2019. *Bacteroidota* occurred at higher percentages in samples collected in 2018 (range 20.1%−26.2%) than in 2019 (average relative abundance ranging from 0.5 to 12.3%; Figure 6).

At order level, members of *UBA10353 marine group* and *Pseudomonadales* were the most abundant in all *H. scotti* specimens (Supplementary Table S4). The former accounted in the range 32.3%−39.6% in 2018, and in the range 28.9%−54.9% in 2019. *Pseudomonadales* showed relative abundances comprised between 13.8 and 17.3%, with higher abundance in individuals collected in 2019 (range 37.4%−43.1%) than 2018. *Flavobacteriales* were more abundant in individuals collected in 2018 (25.4%−26.8%), as well as *Rickettsiales* and *SAR11 clade*, even if they were characterized by relative abundance lower than 10% (Figure 7B).

*Haliclona scotti* specimens differed at genus level in 2018 and 2019 (Figure 8). *Polaribacter* (within *Bacteroidota*), *Pseudohongiella* (within *Gammaproteobacteria*), *Sulfitobacter* (within *Alphaproteobacteria*), LS-NOB (within *Nitrospinota*) were particularly abundant in 2018 specimens, whereas *Pseudohongiella* (within *Gammaproteobacteria*) and *Polaribacter* (within *Bacteroidota*) predominated in 2019.

The Archaeal community of *H. scotti* was exclusively composed of *Candidatus Nitrosopumilus* members (Group 1a *Nitrososphaerota*, formerly *Thaumarchaeota*), with the exception of the specimen *H. dancoi* B1 that showed the sole presence of *Methanothermobacter* (among *Euryarchaeota*) sequences as archaeal representatives.

#### Abiotic matrices

**Figure 9:**
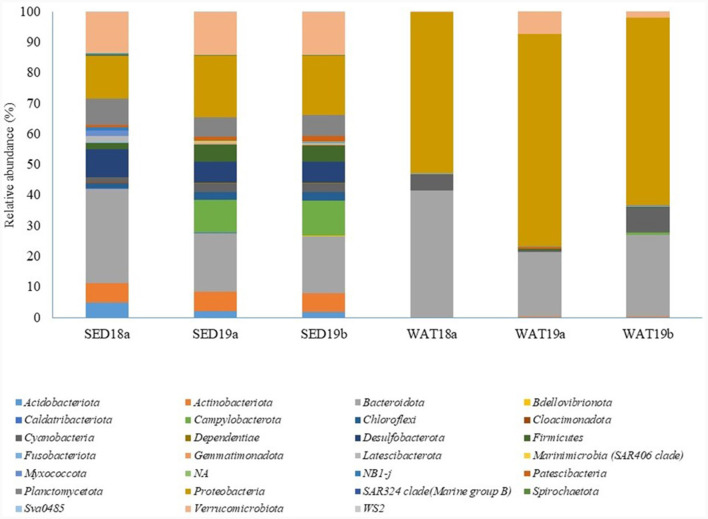
Taxonomic composition of the bacterial communities in sediment and seawater samples collected from the Tethys Bay (Antarctica).

As it was observed for sponges, sediment samples collected in 2018 hosted a bacterial community dominated by *Bacteroidota* and *Proteobacteria* (30 and 14%, respectively), followed by *Verrucomicrobiota* (relative abundance 13.0%), *Desulfobacterota* (9.0%) and *Plancomycetota* (8.7%). The bacterial communities in seawater samples were mostly composed of *Proteobacteria* (relative abundance 52.7%), *Bacteroidota* (41.4%) and, at a lesser extent, *Cyanobacteria* (5.4%; Figure 9).

In line with results obtained for the bacterial communities associated with the two sponge species, bacterial communities inhabiting sediment and seawater samples collected in 2019 were dominated by *Proteobacteria* and *Bacteroidota*. *Proteobacteria* accounted for 20.0 and 19.3% in the two sediment sample replicates, respectively, and for 69.3 and 61.2% in the two seawater replicates, respectively. *Bacteroidota* showed abundances of 19.1 and 18.7% in the two sediment sample replicates, respectively, and of 21.1 and 26.8% in the two seawater sample replicates, respectively. However, some taxa detected in sediment samples were absent or represented at lower abundances in sponge tissues, i.e., *Campylobacterota* (relative abundance 10.6 and 11.5% in SED19a and SED19b, respectively), *Desulfobacterota* (7.1 and 6.8% in SED19a and SED19b, respectively), *Firmicutes* (5.6 and 5.4% in SED19a and SED19b, respectively) and *Planctomycetota* (6.4 and 6.8% in SED19a and SED19b). Conversely, *Cyanobacteria* were retrieved quite exclusively in water samples (relative abundance 8.1% in WAT19b; Figure 9).

At order level, all sediment samples were dominated by *Verrucomicrobiales* and *Flavobacteriales*, with averaged relative abundances comprised between 11.5 and 13.3% and between 17.0 and 27.1%, respectively. *Desulfobacterales* (abundance range 1.5%−3.7%), *Desulfobulbales* (abundance range 5.1%−5.3%), *Pirellulales* (abundance range 4.9%−7.4%) were also detected in sediment samples, but at a lower extent. The presence of *Campylobacterales* was exclusively observed in sediment samples collected in 2019 (relative abundance 10.6 and 11.5%). *Rhodobacterales* (14.5%−36.6%), *Pseudomonadales* (range 14.8%−29.2%) and *Flavobacteriales* (range 21.0%−38.8%) were particularly abundant in seawater sediment (Supplementary Table S4).

No archaeal taxa were detected in water samples from 2018. Conversely, archaeal communities were represented by *Euryarchaeota* and *Halobacterota* in water samples from 2019, with a relative abundance of 69.2 and 30.8% on the total archaeal community, respectively.

Sediment archaeal communities were composed by *Nanoarchaeota* (relative abundance 78.6% of total archaeal community), *Euryarchaeota* (5.7%), and *Crenarchaeota* (2.8%) in sample from 2018. *Nanoarchaeota* and *Euryarchaeota* (accounting for 58.6 and 41.4% of the total archaeal community, respectively) predominated in sediment sampled in 2019.

#### Abiotic vs. biotic matrices

**Figure 10:**
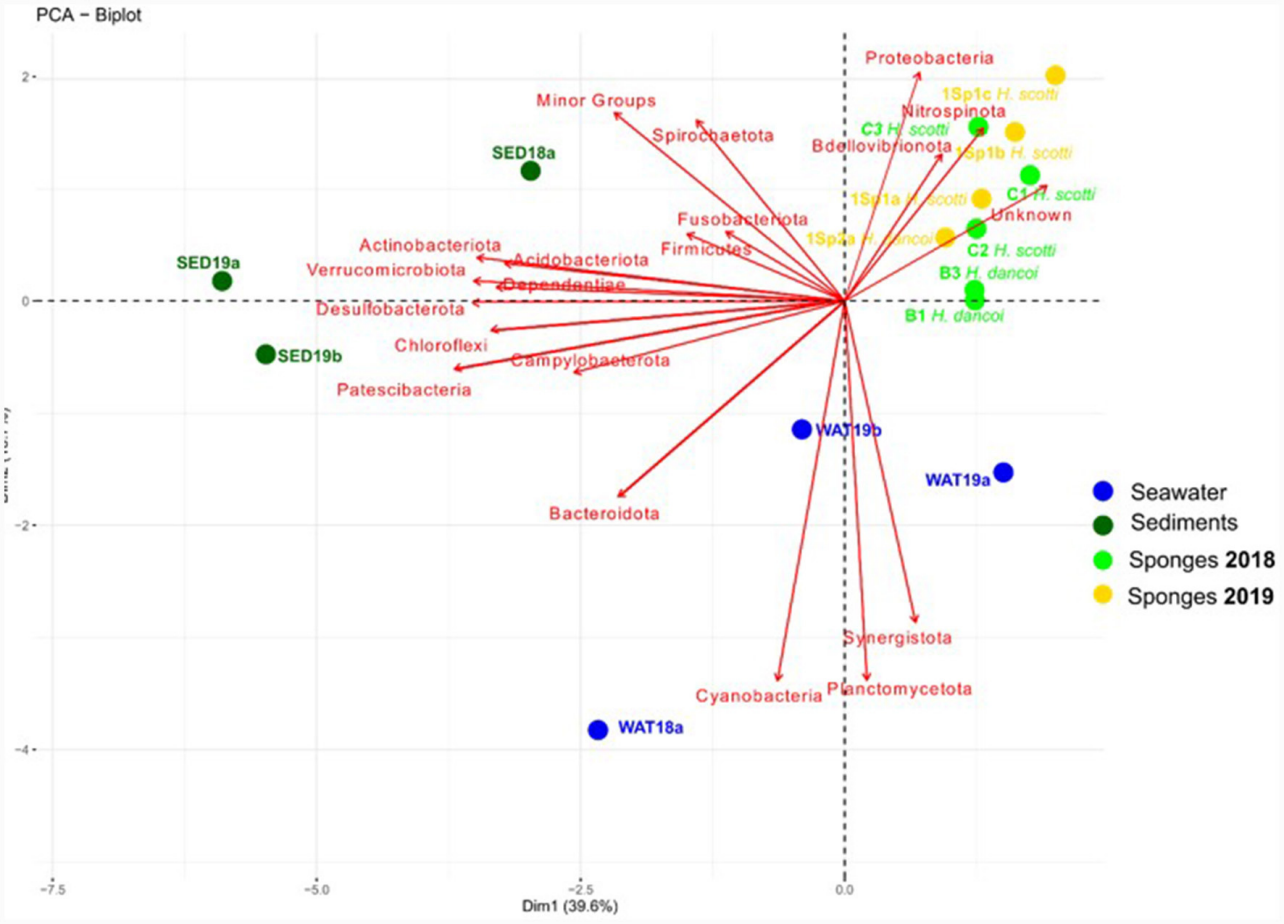
Principal component analysis computed on bacterial community composition in sediment and seawater samples collected from the Tethys Bay (Antarctica).

The statistical analysis performed by PCA (Figure 10) showed a separation of sediment, water and sponge samples depending on the taxonomic composition of the bacterial communities at phylum level. Sediment samples grouped together and showed a positive correlation with *Desulfobacterota, Campylobacterota, Verrucomicrobiota* and *Acidobacteriota*, while water samples resulted more related to *Cyanobacteria* and *Planctomycetota*. Finally, sponges were mainly related to *Nitrospinota, Proteobacteria, Bdellovibrionota* and unknown sequences. The two sponge species clustered separately according to species and year of collection. Indeed, a first cluster was composed of all sponges collected in 2018, with *H. dancoi* B1 and B3 bacterial communities more related to *Firmicutes* and *Actinobacteriota*, and *H. scotti* C1, C2, and C3 bacterial communities more related to *Bacteroidota, Synergistota* and *Desufobacterota* (this latter was exclusively present in *H. scotti* C2). A second large cluster was formed by all samples collected in 2019, even if the single specimen of *H. dancoi* 1Sp2a was separated from the three *H. scotti* individuals, probably due to the higher abundances of some taxonomic groups, such as *Dependentiae, Plancomycetota* and *Campylobacterota*.

### Pollutant level vs. prokaryotes

**Figure 11:**
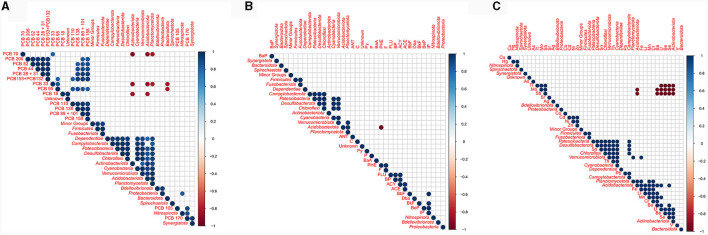
Correlation matrix between the relative abundance of each bacterial phylum and chemical pollutants: **(A)** PCBs; **(B)** PAHs; **(C)** trace metals.

Correlations between the relative abundances detected at phylum level and pollutant concentrations were calculated (Figure 11). A strong negative correlation was detected between phenanthrene concentration and *Acidobacteriota* abundance (*p* ≤ 0.001), while *Actinobacteriota, Acidobacteriota, Planctomycetota*, and *Bacteroidota* were negatively correlated with low chlorinated PCB congeners (*p* ≤ 0.05). Strong negative correlations were detected between *Acidobacteriota* and both Sb and Mo, as well as between *Actinobacteriota* and Sb, Mo and Tl. A positive correlation was generally observed between bacterial phyla and trace metal concentration. For instance, *Cyanobacteria* positively correlated with both Ba and Fe, while *Verrucomicrobiota* positively correlated with both V and U. *Nitrospinota* was the sole phylum showing a significative positive correlation with PCB (namely PCB170). No positive correlations were generally observed between bacteria and PAHs.

**Figure 12:**
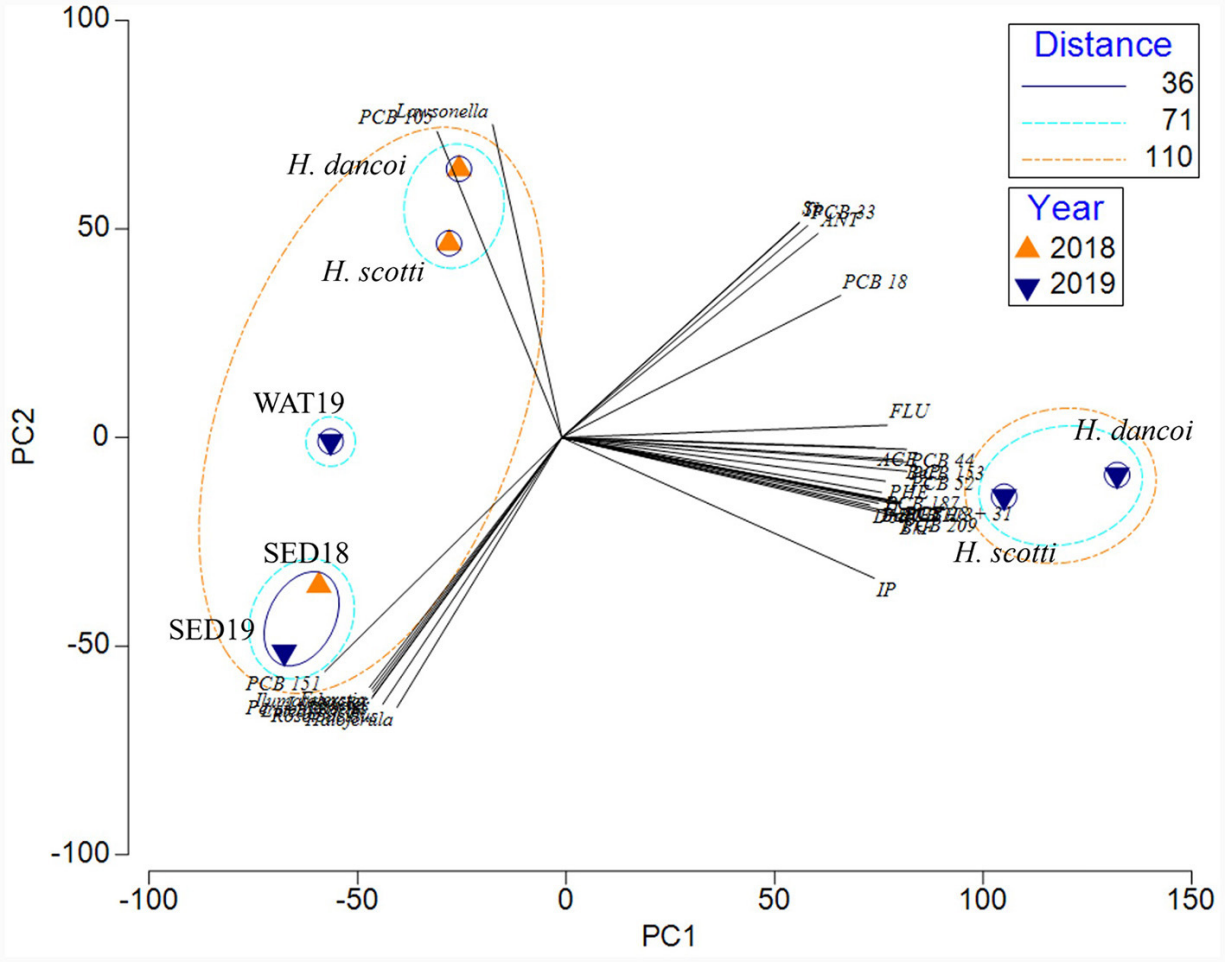
Principal component analysis computed on the entire dataset, including results from chemical and microbiological results (in terms of relative abundance of bacterial taxa at genus level).

Finally, a separation of samples between abiotic and biotic matrices was shown by the PCA (Figure 12). The two principal components explained the 87.5% of the total variance, with PC1 and PC2 accounting for 69.7 and 17.8% of the variance, respectively. The overlapping of the vectors is related to the parameters with a Pearson correlation factor >0.9. Specifically, a separate cluster included the two sponge samples collected in 2019, which were more correlated with the concentration of PCBs and PAHs. A bigger cluster was constituted by three subclusters: the first including the sponge samples collected in 2018, which were more correlated with the presence of both PCB105 and higher abundances of *Lawsonella* sequences; the second one including sediment samples, which were more correlated with the presence of both PCB151 and *Ilumatobacter, Haloferula*, and *Roseibacillus* sequences. Water samples clustered individually.

## Discussion

Even in remote areas, such as Antarctica, biological communities are subjected to stressors of anthropogenic origin. These include pollution which may derive from tourism and research activities in Antarctica (i.e., occurring trough local discharges and emissions), as well as from the long-range transport via atmosphere of pollutants produced at lower latitudes. Porifera are among the first filter-feeding animals that have been recognized as suitable pollution sentinel for tracking trends in anthropogenic contamination in marine coastal waters (Batista et al., 2014). In fact, they have all the requirements needed to be considered bioindicator species, such as their high abundance and wide distribution, sessile and long life, and high tolerance to a variety of environmental factors (Krikech et al., 2022). In this study, two Antarctic sponge species within the genus *Haliclona* (namely *H. scotti* and *H. dancoi*) were characterized for the accumulation of POPs (PCBs and PAHs) and trace metals, with respect to their bulk environment. To the best of our knowledge, in contrast to *H. dancoi* and other non-described *Haliclona* spp., which was previously analyzed for biological and chemical traits and are widespread in Antarctica, *H. scotti* has never been analyzed for the associated microbial community nor for the pollutant content.

Sediments are commonly considered as a valid environmental indicator for the monitoring of pollution of water and marine organisms due to the settling of suspended particulates on the bottom. However, in this study, PCBs and PAHs resulted highly enriched in both sponge species (both in 2018 and 2019) in comparison to sediment (and seawater) samples, suggesting that sponges might be used as pollution indicators even in Antarctica. A variable trend was observed in the case of trace metals, which were often more concentrated in sediment than in sponges or showed concentrations similar to those determined in at least one sponge species. Overall, the different bioaccumulation patterns of tested contaminants determined in *H. scotti* and *H. dancoi* contextually collected from the same sampling site were most likely associated to inter-specific differences in their morphological and physiological traits. Conversely, the differences encountered in pollutant concentrations determined in 2018 and 2019 might be dependent on environmental features that differently characterized both the inner and outer parts of the Thetys Bay at sampling time. These aspects need to be further investigated to individuate mechanisms involved in both pollutant accumulation and excretion processes carried by sponges, and in pollutant accumulation by sponges over time and space.

Vetter and Janussen (2005) first analyzed five species of Antarctic sponges (*Kirkpatrickia variolosa, Artemisina apollinis, Phorbas glaberrima, Halichondria* sp., and *Leucetta antarctica*) from the King George Island to determine the presence of halogenated compounds. PCBs were not detected, whereas lindane, p,p′-DDE and alpha-HCH, in decreasing order of abundance, were detected in traces. The bioaccumulation of PCBs, alongside other POPs (i.e., hexachlorobenzene, HCB, and dichlorodiphenyltrichloroethane, DDT) was recently reported by Pala et al. (2023) in 25 Antarctic sponge specimens collected from the Terra Nova Bay in 2005. Unfortunately, the authors did not analyze sediment or seawater for the determination of pollution level also in the environment surrounding the analyzed sponges. Among the seven sponge specimens collected from the Thetys Bay and analyzed by Pala et al. (2023), a unique specimen (namely TB6) belonged to *H. dancoi*. The authors reported a concentration pattern (i.e., ∑PCB > ∑DDT > HCB) which was common to all samples. Overall, the concentration of most PCB congeners in *H. dancoi* (pg/g of dry weight) determined in our study resulted generally lower than those previously reported by Pala et al. (2023) for the same species; range 70–530 pg/g. Exception was, for example, PCB105 (1,1′-biphenyl, 2,3,3′,4,4′-pentachloro-) that was below the detection level in Pala et al. (2023), but instead accounted for 23.3 pg/g in *H. dancoi* analyzed in our study. However, even if we recorded an increase in PCBs in the 2019 samples, compared to those of 2018 (possibly in dependence on the different sampling sites, i.e., the inner and outer bay), this result gives rise to hope that the banning of the production of PCBs in the 1970s has determined a decreasing trend in their transport and bioaccumulation at higher latitudes. According to Pala et al. (2023), the long-range atmospheric transport was the major driver for POP contamination in the investigated area. However, in a global change scenario several additional factors (e.g., wildlife amplification and ice-melting), in addition to the ever-increasing human presence and activities in Antarctica (e.g., research, tourism and fishing), should be also taken into consideration in the future to individuate those factors that synergistically could stress benthic communities.

PAHs occurring in the marine environment derive from anthropogenic sources. A number of studies have targeted sediment or marine species different from sponges (i.e., mollusks and fish) for the accumulation of PAHs (e.g., Negri et al., 2006; Curtosi et al., 2009; Palmer et al., 2022). To the best of our knowledge, the present study represents the first record on PAH accumulation in Antarctic sponges. PAH concentrations found in sponges were detectable; only anthracene and benzo[a]pyrene were below detection limits and total PAH concentrations ranged from 29.7 to 41.6 mg/g dry weight. All samples had a higher proportion of the lighter (2- to 3-ring) parent PAHs (>63%), with naphthalene and methylnaphthalene being detected in all samples and having the highest concentration in sponge samples. This high proportion of lighter PAHs is probably due to the sponge greater uptake directly from the filtered water and to the higher solubility of lighter PAHs. The complete absence of anthracene, along with the non-negligible presence of phenanthrene (as well as the higher concentration of pyrene compared to fluoranthene), for both sponges leads to the attribution of the origin of the aromatic hydrocarbons to pyrolytic sources and, therefore, to long-term transport radius. Total PAH (2- to 6-ring parent and alkylated) concentrations in the sediment samples collected were around 1–2 μg/kg dry weight.

Heavy metals can reach the Antarctic marine biosphere through four main processes, i.e, long range atmospheric transport and deposition, weathering, biological transportation (i.e., seabird and penguin guanos), and anthropogenic activities (Webb et al., 2020). High concentrations of heavy metals were previously determined in several Antarctic benthic invertebrates, including sponges (Palmer et al., 2022). According to Illuminati et al. (2016), the accumulation of heavy metals (namely cadmium, lead and copper) was significantly lower in the spicules (even if they represent about 80% of the sponge mass) than in the corresponding organic fraction of the Antarctic sponges *Sphaerotylus antarcticus, Kirkpatrikia coulmani* and *Haliclona sp*. *Tedania charcoti* contained zinc and cadmium at remarkable amounts (5,100 and 15,000 mg/kg of dry weight, respectively; Capon et al., 1993). An unidentified sponge from the Antarctic Peninsula contained cadmium, zinc and copper at the a concentration of 3.7, 37, and 3.2 mg/kg of wet weight, respectively (de Moreno et al., 1997). Bargagli et al. (1996) reported cadmium up to 80 mg/kg in *Rossella* sp., *Tedania*, sp. and *Axociella* sp. Such concentrations were generally lower than those determined in our *Haliclona* samples, suggesting an enrichment of certain trace metals in the Thetys Bay area over the time. In our study, at least one *Haliclona* species generally contained amounts of certain heavy metals similar to that determined in sediment. This finding was in line with Negri et al. (2006), who observed comparable amounts of heavy metals in sediment and *Homaxinella balfourensis, Mycale acerata* and *Sphaerotylus antarcticus* from Mc Murdo. Negri et al. (2006) found that only cadmium accumulated to higher concentrations in sponge tissue than in sediments. In our study, in addition to cadmium, also arsenic (both sponge species), nickel and zinc (in *H. dancoi* only), and mercury (in *H. scotti* only) showed a similar trend.

The sponge-associated bacterial community composition strongly differed from those retrieved in both sediment and seawater, suggesting that a sponge-specific bacterial communities could occur in the analyzed species. This finding was in line with previous observations (e.g., Moreno-Pino et al., 2020; Sacristán-Soriano et al., 2020). Furthermore, in accordance with main data available for other Antarctic sponges (e.g., Rodríguez-Marconi et al., 2015; Cárdenas et al., 2018, 2019; Steinert et al., 2019), both sponge species hosted bacterial communities dominated by *Proteobacteria* and *Bacteroidota*, followed by *Firmicutes* and *Planctomycetota*, at phylum level. For instance, a specimen of *H. dancoi* (namely THB8) collected in 2005 from the Thetys Bay hosted a bacterial community dominated by *Alpha*- and *Gammaproteobacteria* (29.6 and 40.1% of the total sequences, respectively), whereas *Actinobacteriota* (similarly to the present study) and *Bacteroidota* accounted for 4.9 and 4.4%, respectively (Papale et al., 2020). Conversely, no data are available on the prokaryotic community specifically associated with *H. scotti* (it was collected from the Thetys Bay more than 114 years after its original description), thus our data representing a baseline.

Consistently with Sacristán-Soriano et al. (2020), archaeal sequences (rarely targeted within the Antarctic sponge-associated prokaryotic communities) were quite exclusively related to *Thaumarchaeota* in both sponge species. Exception was the specimen *H. dancoi* B1 that hosted only *Euryarchaeota*. Among *Thaumarchaeota*, the abundance of the *Candidatus Nitrosopumilus* confirmed previous observations by Moreno-Pino et al. (2020) on the Antarctic sponges *Myxilla* sp. and *Leucetta antarctica*, highlighting its probable role in ammonia oxidation within the host tissues. Finally, the archaeal communities in the targeted sponges were in sharp contrast with those retrieved in sediment and seawater samples, characterized by the overall predominance of *Nanoarchaeota* and *Euryarchaeota*, and the absence of *Thaumarchaeota*.

Notably, despite their high similarities at phylum level, the bacterial communities of *H. scotti* and *H. dancoi* were differently structured at both order and genus level, suggesting the possible selection by the host species, in concomitance or not with environmental factors, of associated microbes, as it was previously observed (e.g., Cárdenas et al., 2019; Sacristán-Soriano et al., 2020). For instance, among main orders, *Bacteroidales, Lactobacillales, Enterobacterales, Burkholderiales*, and *Bacillales* were exclusively, or almost exclusively, associated with *H. dancoi*. The same was true for the UBA10353 marine group, previously reported in association with sponges (i.e., Georgieva et al., 2020; Laroche et al., 2021), that was particularly abundant only in *H. scotti*. Finally, *Flavobacteriales* and *Pseudomonadales* were significantly more abundant in *H. scotti* than *H. dancoi*, whereas *Propionibacteriales* showed an opposite trend. Genera showed a patched distribution among analyzed specimens. However, some of them resulted absent in one or the other species. For instance, *Profundimonas* and *Bdellovibrio* were hosted only by *H. scotti* (both in 2018 and 2019). Conversely, *Bifidobacterium, Lactococcus, Prevotella* and *Porphyromonas* occurred only in association with *H. dancoi*. A further insight into the diversity of sponge-associated prokaryotes, analyzing a higher number of sponge specimens sampled across time and space, is needed to elucidate the interactions between microbes and their Antarctic benthic hosts, by establishing if the observed exclusive phylotypes are actual members of the sponge core microbiomes of *H. dancoi* and *H. scotti*, and to individuate sponge intrinsic features driving the observed specific association.

Microbes associated with sponges are thought to thrive with the occurrence of organic and inorganic pollutants in their host tissues, possibly protecting sponges by transforming pollutants or participating in their excretion, as it was supposed by Perez et al. (2003) for some PCB congeners in the Mediterranean *Spongia officinalis*. The potential of sponge-associated bacterial communities in degrading aromatic compounds was recently suggested by the application of predictive functional analyses on 16S rRNA gene data (Steinert et al., 2019; Moreno-Pino et al., 2020; Papale et al., 2020; Cristi et al., 2022). Mangano et al. (2014) observed that bacterial isolates from the Antarctic sponge *Hemigellius pilosus* showed resistance to cadmium, suggesting that this probably allowed them to gain residence in the host tissue. Later, the ability to tolerate high heavy metal concentration (i.e., mercury and cadmium), was demonstrated in exopolysaccharide-producing bacterial isolates from the Antarctic sponges *Haliclonissa verrucosa, H. pilosus* and *T. charcoti* (Caruso et al., 2018). It is not to be excluded that the different levels in the bioaccumulation of tested contaminants observed in *H. scotti* and *H. dancoi* from the same sampling site might drive by the bacterial communities associated with the sponge host, in dependence of their toxicity, as well as bacterial resistance and/or transformation. In our study, the statistical analyses, aimed at comparing microbiological and chemical data in sponge tissues, allowed us to suppose that pollutant levels in Antarctic sponges could be a sponge non-biological feature involved in the establishment of associated bacterial communities. For instance, at phylum level, the low abundance of *Acidobacteriota* and *Actinobacteriota* might be dependent on the concentration of certain pollutants (e.g., phenanthrene, low chlorinated PCBs and some trace metals, such as molybdenum, antimony and thallium) in the sponge tissues. Conversely, *Nitrospinota* was the sole phylum showing a significative positive correlation with PCB (namely PCB170). To date, the study of the effects of pollution on microbes in their natural environment has been very limited and mainly addressed to soils (especially agricultural fields) and sediments. A numerical reduction of *Acidobacteria* in the presence of phenanthrene was previously reported in soil microbial communities (Sipila et al., 2008; Ding et al., 2012). Furthermore, Festa et al. (2018) observed that *Actinobacteria* and *Acidobacteria* were significantly repressed in phenanthrene-amended microcosms. *Acidobacteria*, together with *Proteobacteria* and *Firmicutes*, are generally reported as bacterial phyla associated with PCB contaminated sediments (Zenteno-Rojas et al., 2020). Among *Proteobacteria*, the ammonia-oxidizing *Nitrosococcales* (*Gammaproteobacteria*) positively correlated with both PCBs and PAHs, whereas the congener PCB105 (i.e., 1,1′-biphenyl, 2,3,3′,4,4′-pentachloro-) correlated to the bacterial orders *Defluvicoccales* (within *Alphaproteobacteria*) and *Coxiellales* (within *Gammaproteobacteria*). Among *Actinobacteria*, PCB105 positively correlated also to the order *Micromonosporales*. In addition, the PCA showed a strong relationship between PCB105 and the genus *Lawsonella* from both sponges. Further investigations should be addressed to the isolation of members of these genera to be tested for the degradation of selected PCB congeners. Unlike our results, the exposure to PCB congeners and Aroclor 1242 resulted in the selection of bacterial groups belonging to potential PCB degraders, i.e., *Betaproteobacteria* and *Acidobacteria*, with a decrease of toxicity with increased chlorine substitution (Correa et al., 2010; Nuzzo et al., 2017). Differently from PCBs and PAHs, trace metals seemed to favor the occurrence of *Cyanobacteria* and *Verrucomicrobiota*, suggesting their tolerance to metals occurring in the sponge mesohyl tissues. Overall, results from our study did not allow to discern specific patterns linking the exposure of sponge-associated bacterial communities to pollutants and the different bacterial community structures. Experiments in microcosm with Antarctic sponges exposed to pollutants (individual or combined) could be performed to disentangle bacterial community dynamics over time.

## Concluding remarks

This study allowed obtaining important information on the bioaccumulation of a selection of persistent organic pollutants (i.e., PCBs and PAHs) and trace metals, along with the composition of the associated prokaryotic communities, in the Antarctic sponge species *H. scotti* and *H. dancoi*. In particular, we report for the first time on microbiological and chemical features of *H. scotti*, representing a rare species in the Ross Sea. The accumulation of the targeted inorganic and organic contaminants by the two sponge species appeared evident, as it was demonstrated by their lower concentrations in abiotic matrices (i.e., sediment and seawater), which surrounded sponge individuals at sampling time. Overall, in comparison with previous investigations, we observed an increased concentration of trace metals and, conversely, a decrease in the level of PCBs in the sponge tissue. Moreover, the analysis of PAHs in Antarctic sponges reported for the first time in this study. From a microbiological point of view, our finding confirmed previous observations on the predominance of *Proteobacteria* and *Bacteroidota*, as well as the low abundance of *Archaea*, within the prokaryotic communities associated with Antarctic sponges, with some bacterial traits (mainly at order and genus levels) resulting sponge-species specific.

Results obtained in this study represent a baseline for further investigations aimed at disentangling the interactions between prokaryotes and Porifera in the Antarctic environment. The obtained outcomes suggest to take into consideration in future research anthropogenic stress factors in addition to biological features. This is the case of pollution level, that directly or indirectly could increase in polar areas following ice-melting with consequent release of contaminants entrapped for a long time within glaciers, affecting the biota. Further studies carried out under controlled conditions and targeting selected pollutants and bacterial taxa are certainly needed to elucidate both the pollutant bioaccumulation rate by Antarctic sponges and the actual effect of contamination in structuring the sponge-associated prokaryotic communities. Finally, testing bacterial isolates for POP degradation capability and efficiency, in addition to metal tolerance, could furnish further information on the adaptation of bacteria to the sponge environment and their role in the protection of their host.

## Data availability statement

The datasets presented in this study can be found in online repositories. The names of the repository/repositories and accession number(s) can be found in the article/Supplementary material.

## Ethics statement

Ethical approval was not required for the study involving animals in accordance with the local legislation and institutional requirements because it was not required as invertebrates were used in this study.

## Author contributions

MP: Data curation, Investigation, Methodology, Software, Writing – original draft, Writing – review & editing. SG: Data curation, Investigation, Methodology, Writing – review & editing. MA: Investigation, Methodology, Writing – review & editing. LG: Data curation, Investigation, Writing – review & editing. AL: Conceptualization, Data curation, Funding acquisition, Supervision, Writing – original draft, Writing – review & editing. CR: Data curation, Investigation, Methodology, Software, Writing – original draft, Writing – review & editing.
