# Supplementary material for: Chemical and microbiological insights into two littoral Antarctic demosponge species: Haliclona (Rhizoniera) dancoi (Topsent 1901) and Haliclona (Rhizoniera) scotti (Kirkpatrick 1907)
Source: Front Microbiol. 2024 Feb 9;15:1341641. doi: 10.3389/fmicb.2024.1341641 (PMC10884823; doi:10.3389/fmicb.2024.1341641)
Supplement: Supplementary file 1 [file Table_1.DOCX]

Supplementary Material

Chemical and microbiological insight into two littoral Antarctic demosponge species: *Haliclona* (*Rhizoniera*) *dancoi* (Topsent 1901) and *Haliclona* (*Rhizoniera*) *scotti* (Kirkpatrick 1907)

Maria Papale^1^, Stefania Giannarelli^2^, Maurizio Azzaro^1^, Lisa Ghezzi^3^, Angelina Lo Giudice^1*^, Carmen Rizzo^1,4^

^1^ Institute of Polar Sciences, National Research Council, Messina, Italy

^2^ Department of Chemical and Industrial Chemistry, University of Pisa, Pisa, Italy

^3^ Department of Earth Sciences, University of Pisa, Pisa, Italy

^4^ Zoological Station “Anton Dohrn”, Messina, Italy

*** Correspondence:**Angelina Lo Giudice
[angelina.logiudice@cnr.it](mailto:angelina.logiudice@cnr.it)

Keywords: *Haliclona* spp., persistent organic pollutants, heavy metals, prokaryotic communities, Antarctic Porifera

**Supplementary Table S1.** Retention time (RT) and selected transitions.

**Supplementary Table S2.** Pollutant concentrations in sponge, sediment and seawater samples collected from the Thetys Bay. nq: not quantified (conc.> LOD and < LOQ); nd: not detected (conc < LOD). na: not analyzed.

**Supplementary Table S3.** Main data on total sequence reads, quality trimming, ASV information and diversity indices obtained for samples included in this study.

**Supplementary Table S4.** Main data on ASV relative abundance at order level for samples included in this study.

**Supplementary Table S1.** Retention time (RT) and selected transitions.

**Compound (min) QUANTIFIER ION (M/Z) QUALIFIER ION (M/Z) RT (min)**

Acenaphthylene ACY 152.1⟶126.0 152.1⟶102.1 7.8

Acenaphthene ACE 152.1⟶126.0 153.1⟶127.0 8.2

Fluorene FLU 166.1⟶165.1 165.1⟶164.1 9.5

Phenanthrene PHE 178.1⟶152.1 176.1⟶150.1 12.6

Anthracene ANT 178.1⟶151.1 178.1⟶152.1 12.9

Fluoranthene F 201.1⟶200.1 202.1⟶152.1 17.5

Pyrene Py 201.1⟶200.1 200.1⟶174.0 18.4

Benzo [a] Anthracene BaA 228.1⟶226.1 114.0⟶101.1 23.9

Chrysene C 228.1⟶226.1 113.1⟶112.1 24.1

Benzo [b] Fluoranthene BbF 252.1⟶250.1 126.0⟶113.1 28.5

Benzo [k] Fluoranthene BkF 252.1⟶250.1 126.1⟶113.1 28.7

Benzo [e] Pyrene BeP 252.1⟶250.1 126.1⟶113.1 29.6

Benzo [a] Pyrene BaP 252.1⟶250.1 125.0⟶124.1 29.8

Perylene P 252.1⟶250.1 126.0⟶125.1 30.1

Benzo [g,h,i] Perylene BP 137.0⟶136.0 138.0⟶137.0 33.8

Dibenzo[a,h]anthracene DBA 278.1⟶276.1 125.0⟶124.1 34.0

Indeno [1,2,3-c,d] Pyrene IP 137.0⟶136.0 138.1⟶137.1 34.6

2,2',5-Trichlorobiphenyl PCB 18 221.0⟶186.0 226.0⟶186.0 12.7

4,4'-Dichlorobiphenyl PCB 15 222.0⟶152.0 224.0⟶152.1 12.8

2,4,4'- Trichlorobiphenyl PCB 28 256.0⟶186.0 258.0⟶186.0 14.3

2',3,4- Trichlorobiphenyl PCB 33 256.0⟶186.0 258.0⟶186.0 14.7

2,2',5,5'- Tetrachlorobiphenyl PCB 52 289.9⟶219.9 255.0⟶220.0 15.7

2,2',3,5'- Tetrachlorobiphenyl PCB 44 289.9⟶219.9 255.0⟶220.0 16.3

2,3',4',5'- Tetrachlorobiphenyl PCB 70 289.9⟶219.9 291.9⟶221.9 17.7

[2,2′,3,5′,6](https://pubs.acs.org/doi/10.1021/acs.chemrestox.6b00371)**-** Pentachlorobiphenyl PCB 95 250.9⟶184.0 325.9⟶255.9 17.8

2,2',4,5,5'- Pentachlorobiphenyl PCB 89 253.9⟶184.0 325.9⟶255.9 18.7

2,2',4,4',5- Pentachlorobiphenyl PCB 99 250.9⟶184.0 325.9⟶184.0 18.8

2,3,3',5',6- Pentachlorobiphenyl PCB 113 250.9⟶184.0 325.9⟶184.0 18.8

2,3,3',4',6- Pentachlorobiphenyl PCB 110 253.9⟶184.0 325.9⟶255.9 19.9

2,2',3,5,5',6- Hexachlorobiphenyl PCB 151 359.9⟶289.9 359.9⟶324.9 20.4

2,2',3,4',5',6- Hexachlorobiphenyl PCB 149 359.9⟶289.9 287.9⟶217.9 20.8

2,3',4,4',5- Pentaclorobifenile PCB 118 325.9⟶255.9 325.9⟶253.9 20.9

2,2',4,4',5,5'- Hexachlorobiphenyl PCB 153 359.9⟶289.9 287.9⟶217.9 21.7

2,3,3',4,4'- Pentaclorobifenile PCB 105 253.9⟶184.0 325.9⟶255.9 21.8

2,2',3,3',4,6'- Hexachlorobiphenyl PCB 132 359.9⟶289.9 359.9⟶324.9 21.8

2,2',3,4,4',5'- Hexachlorobiphenyl e PCB 138 359.9⟶289.9 287.9 ⟶ 217.9 22.6

2,2',3,4',5,5',6- Heptachlorobiphenyl PCB 187 393.8⟶358.8 393.8⟶323.8 23.2

2,2',3,3',4,4'- Hexachlorobiphenyl PCB 128 359.9⟶289.9 287.9⟶217.9 23.6

2,3,3',4,4',6- Hexachlorobiphenyl PCB 158 359.9⟶289.9 361.9⟶289.9 23.6

2,3,3',4,4',5- Hexachlorobiphenyl PCB 156 359.9⟶289.9 361.9⟶289.9 24.4

2,2',3,4,4',5,5'- Hexachlorobiphenyl PCB 180 393.8⟶358.8 393.8⟶323.8 25.0

2,2',3,3',4,4',5- Heptachlorobiphenyl PCB 170 393.8⟶323.8 393.8⟶358.8 26.0

2,2',3,3',4,5,5',6'- Octachlorobiphenyl PCB 199 427.8⟶392.8 429.8⟶392.8 26.3

**Compound (min) QUANTIFIER ION (M/Z) QUALIFIER ION (M/Z) RT (min)**

2,2',3,3',4,4',5,6- Octachlorobiphenyl PCB 195 427.8⟶392.8 429.8⟶394.8 27.4

2,2',3,3',4,4',5,5'- Octachlorobiphenyl PCB 194 427.8⟶357.8 427.8⟶392.8 28.1

2,2',3,3',4,4',5,5',6- Nonachlorobiphenyl PCB 206 461.8⟶391.7 463.8⟶393.7 29.3

Decachlorobiphenyl PCB 209 497.7⟶427.7 495.8⟶425.7 30.3

**Supplementary Table S2.** Pollutant concentrations in sponge, sediment and seawater samples collected from the Thetys Bay. nq: not quantified (conc.> LOD and < LOQ); nd: not detected (conc < LOD). na: not analyzed.

|  |  | **2018** | | |  | **2019** | | | |
| --- | --- | --- | --- | --- | --- | --- | --- | --- | --- |
| **Compound** | **Code** | ***H. dancoi* B** | ***H. scotti* C** | **Sediment** |  | ***H. dancoi* B** | ***H. scotti* C** | **Sediment** | **Water** |
| **PCBs (pg/g)** |  |  |  |  |  |  |  |  |  |
| 2,2',5-Trichlorobiphenyl (BZ 18) | PCB 18 | 23.5 ± 4.7 | 52.6 ± 10.5 | 1.2 ± 0.2 |  | 65.4± 1.2 | 42.1 ± 0.01 | nq | 4.7 ± 0.5 |
| 4,4'-Dichlorobiphenyl (BZ 15) | PCB 15 | nq | 11.1 ± 2.2 | 1.5 ± 0.3 |  | 16.4± 0.5 | nq | nq | 1.5 ± 0.1 |
| 2,4,4′-Trichlorobiphenyl+2,4′,5-Trichlorobiphenyl (BZ 28 + BZ 31) | PCB 28 + 31 | nq | nq | 3.1 ± 0.6 |  | 208.6± 8.5 | 149.5 ± 0.01 | 0.3 ± 0.1 | 2.9 ± 0.4 |
| 2',3,4-Trichlorobiphenyl (BZ 33) | PCB 33 | 84.2 ± 16.8 | 40.8 ± 8.2 | 3.1 ± 0.6 |  | 83.9± 4,3 | 55.1 ± 0.01 | 0.3 ± 0.1 | 6.5 ± 0.8 |
| 2,2',5,5'-Tetrachlorobiphenyl (BZ 52) | PCB 52 | 13.8 ± 2.8 | 18.7 ± 3.7 | 2.4 ± 0.5 |  | 150.3± 8.5 | 135.6 ± 0.01 | 0.7 ± 0.1 | nq |
| 2,2',3,5'-Tetrachlorobiphenyl (BZ 44) | PCB 44 | 7.5 ± 1.5 | 17.8 ± 3.6 | 1.9 ± 0.4 |  | 70.9± 7.5 | 60.1 ± 0.01 | 0.3 ± 0.1 | nq |
| 2,3',4',5-Tetrachlorobiphenyl (BZ 70) | PCB 70 | 31. 9± 6.4 | 23.5 ± 4.7 | 1.5 ± 0.3 |  | 35.5± 0.5 | 60.5 ± 0.03 | 0.4 ± 0.1 | 2.4 ± 0.3 |
| 2,2',3,5',6-Pentachlorobiphenyl (BZ 95) | PCB 95 | 20.1 ± 4.0 | 2.7 ± 0.5 | 1.4 ± 0.3 |  | 36.5± 0.6 | 8.1 ± 0.0 | 1.1 ± 0.2 | 4.5 ± 0.5 |
|  | PCB 89 + 101 | nd | nd | 1.5 ± 0.3 |  | 218.3± 10.5 | 68 ± 0.01 | nq | nq |
| 2,3,3',5',6-Pentachlorobiphenyl (BZ 113) | PCB 113 | nd | nd | nq |  | 13.9± 0.5 | 16.7 ± 0.01 | nq | nq |
| 2,2',4,4',5-Pentachlorobiphenyl (BZ 99) | PCB 99 | 16.5 ± 3.3 | 2.4 ± 0.5 | 1.6 ± 0.3 |  | nd | nq | 0.9 ± 0.3 | 2.2 ± 0.4 |
| 2,3,3',4',6-Pentachlorobiphenyl (BZ 110) | PCB 110 | nq | 3.9 ± 0.8 | 2.0 ± 0.4 |  | 192.5± 9.8 | nq | 0.9 ± 0.2 | 2.0 ± 0.2 |
| 2,2',3,5,5',6-Hexachlorobiphenyl (BZ 151) | PCB 151 | nq | nq | 4.0 ± 0.8 |  | nd | nq | 3.4 ± 0.7 | 3.1 ± 0.6 |
| 2,2',3,4',5',6-Hexachlorobiphenyl (BZ 149) | PCB 149 | 4.5 ± 0.9 | 5.4 ± 1.1 | 1.1 ± 0.3 |  | 9.0± 0.3 | 27.5 ± 0.05 | 1.1 ± 0.2 | 2.1 ± 0.2 |
| 2,3',4,4',5-Pentachlorobiphenyl (BZ 118) | PCB 118 | 19.7 ± 3.9 | 8.2 ± 0.8 | 1.7 ± 0.3 |  | 0.7± 0.2 | 57.4 ± 0.0 | 1.4 ± 0.3 | 5.0 ± 0.5 |
| 2,2′,4,4′,5,5′-Hexachlorobiphenyl+2,2',3,3',4,6'-Hexachlorobiphenyl | PCB 153+PCB132 | 15.5 ± 3.1 | 9.6 ± 0.9 | 2.8 ± 0.5 |  | 85.3± 6,2 | 77.7 ± 0.05 | 2.9 ± 0.6 | 5.6 ± 0.2 |
| 2,3,3',4,4'-Pentachlorobiphenyl (BZ 105) | PCB 105 | 23.3 ± 4.7 | 25.1 ± 2.2 | 6.4 ± 1.3 |  | nd | nq | nq | 5.1 ± 1.0 |
| 2,2',3,4,4',5'-Hexachlorobiphenyl (BZ 138) | PCB 138 | 14.8 ± 3.0 | 13.8 ± 1.7 | 2.1 ± 0.4 |  | 143.3± 7.5 | 51.2 ± 0.03 | 3.9 ± 0.8 | 5.2 ± 0.2 |
| 2,2',3,4',5,5',6-Heptachlorobiphenyl (BZ 187) | PCB 158 | nd | nd | 1.8 ± 0.4 |  | 54.2± 0.9 | 25.3 ± 0.01 | 2.3 ± 0.5 | nd |
| 2,3,3',4,4',6-Hexachlorobiphenyl (BZ 158) | PCB 187 |  | 7.6 ± 1.5 | 5.2 ± 1.0 |  | nd | 3.5 ± 0.0 | 4.8 ± 1.0 | 3.3 ± 0.7 |
| 2,2',3,3',4,4'-Hexachlorobiphenyl+2,3,3′,4,4′,5-Hexachlorobiphenyl | PCB 128+PCB156 | nq | 12.2 ± 1.5 | nq |  | nd | nq | 6.5 ± 1.3 | nd |
| 2,2',3,4,4',5,5'-Heptachlorobiphenyl (BZ 180) | PCB 180 | 9.7 ± 1.9 | 9.9 ± 1.1 | 5.3 ± 1.1 |  | 6.123 | 3.8 ± 0.0 | 6.1 ±1.2 | 3.6 ± 0.3 |
|  |  | **2018** | | |  | **2019** | | | |
| **Compound** | **Code** | ***H. dancoi* B** | ***H. scotti* C** | **Sediment** |  | ***H. dancoi* B** | ***H. scotti* C** | **Sediment** | **Water** |
| 2,2',3,3',4,4',5-Heptachlorobiphenyl (BZ 170) | PCB 170 |  | *32.2 ± 2.0* | 2.6 ± 0.5 |  | *nq* | *10.6 ± 0.0* | 3.4 ± 0.7 | nd |
| 2,2',3,3',4,5,5',6'-Octachlorobiphenyl (BZ 199) | PCB 199 | nd | nd | 1.9 ± 0.4 |  | nq | nq | 2.1 ± 0.4 | nd |
| 2,2',3,3',4,4',5,6-Octachlorobiphenyl (BZ 195) | PCB 195 | nd | nd | 3.9 ± 0.8 |  | nq | nq | nq | 2.2 ± 0.4 |
| 2,2',3,3',4,4',5,5'-Octachlorobiphenyl (BZ 194) | PCB 194 | nq | nq | 3.8 ± 0.8 |  | nq | nq | 4.0 ± 0.8 | nd |
| 2,2',3,3',4,4',5,5',6-Nonachlorobiphenyl (BZ 206) | PCB 206 | nd | nd | nd |  | 66.7± 1.3 | 60.4 ± 0.01 | 4.8 ± 1.0 | nd |
| Decachlorobiphenyl (BZ 209) | PCB 209 | 2.8 ± 0.6 | nd | nd |  | nq | nq | nd | nd |
|  |  |  |  |  |  |  |  |  |  |
| **PAHs (pg/g)** |  |  |  |  |  |  |  |  |  |
| Naphthalene | NAP | nd | nd | nd |  | 5497.9±0.5 | 5769.2 ± 0.3 | nd | nd |
| Methylnaphthalene | METH | nd | nd | nd |  | 10112.0±0.4 | 9720.6 ± 0.0 | nq | nd |
| Acenaphthylene | ACY | 138.2 ± 27.6 | 125.8 ± 25.2 | nq |  | 1296.8±0.9 | 458 ± 0.1 | nq | nq |
| Acenaphthene | ACE | 368.5 ± 73.7 | 80.0 ± 16.0 | 1.3 ± 0.3 |  | 1432.3±0.4 | 691.2 ± 0.2 | nq | 12.9 ± 0.7 |
| Fluorene | FLU | 217.7 ± 12.9 | 156.6 ± 31.3 | 18.3 ± 3.7 |  | 6333.2±0.7 | 2827.6 ± 0.7 | nq | 58.3 ± 11.7 |
| Phenanthrene | PHE | 1555.9 ± 311.2 | 1708.3 ± 341.7 | 248.8 ± 26.8 |  | 1670.3±0.8 | 1976 ± 0.3 | 22.2 ± 4.4 | 159.0 ± 31.8 |
| Anthracene | ANT | 368.5 ± 73.7 | 141.0 ± 28.2 | nq |  | nd | nq | nq | 9.6 ± 1.0 |
| Fluoranthene | F | 919.5 ± 183.9 | 1174.3 ± 234.9 | 129.4 ± 24.0 |  | 1005.9±1.0 | 1856.9 ± 0.0 | 150.8 ± 30.2 | 31.0 ± 6.2 |
| Pyrene | Py | 2744.0 ± 477.9 | 4083.6 ± 816.7 | 131.3 ± 16.4 |  | 3229.7±0.5 | 3657.6 ± 0.1 | 101.2 ± 20.2 | 39.8 ± 8.0 |
| Benz[a]anthracene | BaA | 465.4 ± 42.2 | 468.2 ± 93.6 | 22.6 ± 4.5 |  | 185.2± 0.1 | 210.8 ± 0.0 | 73.7 ± 14.7 | nd |
| Crysene | C | 3673.9 ± 738.8 | 1936.5 ± 387.3 | 477.3 ± 95.5 |  | 2417.1±0.9 | 921.4 ± 0.5 | 299.8 ± 60.0 | 20.9 ± 4.2 |
| Benzo[b]fluoranthene | BbF | 68.8 ± 13.8 | 128.0 ± 25.6 | 162.6 ± 33.5 |  | 746.2± 1.5 | 679.8 ± 0.0 | 112.4 ± 22.5 | 1.0 ± 0.1 |
| Benzo[k]fluoranthene | BkF | 253.6 ± 50.7 | 68.7 ± 13.7 | 25.5 ± 5.1 |  | 953.7± 1.3 | 967.6 ± 0.0 | 65.5 ± 13.1 | 8.8 ± 1.8 |
| Benzo[e]pyrene | BeP | 58.5 ± 11.7 | 52.8 ± 10.6 | 69.9 ± 14.0 |  | 214.9± 0.7 | 309.2 ± 0.0 | 52.3 ± 10.5 | 2.4 ± 0.4 |
| Benzo[a]pyrene | BaP | 159.0 ± 31.8 | 363.7 ± 72.7 | 13.2 ± 2.6 |  | nd | nq | 67.4 ± 13.5 | 7.8 ± 1.3 |
| Perylene | P | 101.9 ± 20.4 | 233.2 ± 46.6 | 23.8 ± 4.8 |  | 206.8± 0.3 | 60.4 ± 0.0 | 42.8 ± 8.6 | 4.2 ± 0.8 |
| Benzo(ghi)Perilene | BP | nq | nq | 56.7 ± 11.3 |  | 1110.3±0.9 | 406.2 ± 0.0 | 48.3 ± 9.7 | 6.0 ± 0.8 |
| Dibenz[a,h]anthracene | Dba | nq | nq | 33.5 ± 6.7 |  | 114.3±0.5 | 122.9 ± 0.0 | 29.4 ± 5.9 | nd |
| Indeno[1,2,3-cd]pyrene | IP | nq | nq | 124.1 ± 24.8 |  | 5085.6±0.9 | 2194 ± 0.1 | 104.2 ± 20.8 | nd |
|  |  |  |  |  |  |  |  |  |  |
| **Trace metals (ppm)** |  |  |  |  |  |  |  |  |  |
| Mercury (µg/kg) | Hg (ppb) | 61 ± 6 | 450 ± 45 | 9.5 ± 1.0 |  | 93± 10 | 592 ± 193 | 10.3 ± 1.8 | na |
|  |  | **2018** | | |  | **2019** | | | |
| **Compound** | **Code** | ***H. dancoi* B** | ***H. scotti* C** | **Sediment** |  | ***H. dancoi* B** | ***H. scotti* C** | **Sediment** | **Water** |
| Litium | Li | 1.1 ± 0.1 | 1.2 ± 0.1 | 7.1 ± 0.7 |  | 1.4 ± 0.1 | 2.7 ± 1.4 | 10.3 ± 0.1 | na |
| Manganese | Mn | 9.3 ± 0.5 | 7.5 ± 0.4 | 85 ± 4 |  | 2.9 ± 0.1 | 18.8 ± 15 | 120 ± 16 | na |
| Cobalt | Co | 3.2 ± 0.3 | 0.24 ± 0.02 | 1.5 ± 0.1 |  | nd | 0.6 ± 0.2 | 2.4 ± 0.4 | na |
| Nichel | Ni | 705 ± 70 | 2.7 ± 0.3 | 5.2 ± 0.5 |  | nd | 22.8 ± 1.4 | 11.7 ± 4.8 | na |
| Copper | Cu | 6.2 ± 0.6 | 9.4 ± 0.9 | 3.3 ± 0.3 |  | 6.8 ± 0.7 | 11.8 ± 2.0 | 4.3 ± 0.7 | na |
| Zinc | Zn | 1854 ± 185 | 84 ± 8 | 20.6 ± 2.0 |  | nd | 181 ± 18 | 32 ± 2.0 | na |
| Strontium | Sr | 83 ± 8 | 94 ± 9 | 19.1 ± 2.0 |  | 71 ± 7 | 78 ± 27 | 94 ± 34 | na |
| Silver | Ag | 0.48 ± 0.02 | 1.2 ± 0.1 | 0.10 ± 0.01 |  | 0.69 ± 0.03 | 0.5 ± 0.1 | 0.1 ± 0.1 | na |
| Cadmium | Cd | 375 ± 19 | 42 ± 2 | 0.7 ± 0.1 |  | 0.95 ± 8 | 46 ± 17 | 0.8 ± 0.4 | na |
| Barium | Ba | 8.4 ± 0.8 | 8.5 ± 0.8 | 30 ± 3 |  | 4.6 ± 0.5 | 10.9 ± 3.5 | 34 ± 8 | na |
| Vanadium | V | 4.8 ± 0.2 | 8.7 ± 0.4 | 10.3 ± 0.5 |  | 4.4 ± 0.2 | 8.3 ± 1.7 | 12.9 ± 2.2 | na |
| Chrome | Cr | nd | nd | 7.1 ± 0.4 |  | nd | 2.3 ± 0.8 | 10.6 ± 5.5 | na |
| Iron | Fe | 218 ± 22 | 406 ± 41 | 5017 ± 500 |  | 140 ± 14 | 1223 ± 122 | 8765 ± 309 | na |
| Arsenic | As | 16.0 ± 0.8 | 16.3 ± 0.8 | 8.7 ± 0.4 |  | 22.7 ± 1.1 | 17.9 ± 4.7 | 3.3 ± 0.7 | na |
| Thorium | Th | nd | 0.45 ± 0.05 | 3.6 ± 0.4 |  | nd | 0.9 ± 0.1 | 11.2 ± 1.9 | na |
| Uranium | U | 0.14 ± 0.01 | 0.18 ± 0.02 | 0.74 ± 0.07 |  | nd | 0.17 ± 0.04 | 0.8 ± 0.2 | na |
| Beryllium | Be | nd | nd | 0.31 ± 0.02 |  | nd | nd | 0.3 ± 0.1 | na |
| Molybdenum | Mo | nd | nd | 0.54 ± 0.03 |  | nd | nd | 0.5 ± 0.1 | na |
| Tin | Sn | nd | nd | 0.52 ± 0.03 |  | nd | nd | 0.6 ± 0.1 | na |
| Antimony | Sb | nd | nd | nd |  | nd | nd | nd | na |
| Thallium | Tl | nd | nd | 0.07 ± 0.01 |  | nd | nd | 0.1 ± 0.0 | na |
| Lead | Pb | nd | nd | 0.83 ± 0.04 |  | nd | nd | 1.6 ± 0.0 | na |

**Supplementary Table S3.** Main data on total sequence reads, quality trimming, ASV information and diversity indices obtained for samples included in this study.

|  | input | filtered | merged | nonchim | % of Good Reads | Observed ASVs | Chao1 | ACE | Shannon | Simpson | InvSimpson | Fisher |
| --- | --- | --- | --- | --- | --- | --- | --- | --- | --- | --- | --- | --- |
| *H. scotti* 1Sp1a (2019) | 53744.5 | 49108.5 | 48366 | 46371 | 85.66 | 470 | 492.79 | 486.32 | 3.42 | 0.88 | 9.29 | 77.34 |
| *H. scotti* 1Sp1b (2019) | 78952.33 | 57346.67 | 57156.67 | 56119 | 65.35 | 143 | 146.3 | 147.56 | 2.02 | 0.74 | 3.87 | 17.99 |
| *H. scotti* 1Sp1c (2019) | 71834.5 | 66648 | 66066.5 | 58079.5 | 80.4 | 313.5 | 341.43 | 337.62 | 2.49 | 0.84 | 6.32 | 43.88 |
| *H. dancoi* 1Sp2a (2019) | 32337 | 12101 | 11851.5 | 11465.5 | 47.78 | 214 | 219.38 | 219.22 | 4.17 | 0.97 | 41.35 | 37.52 |
| *H. dancoi* B1 (2018) | 22361.67 | 12167 | 12017.67 | 11178.67 | 52.54 | 84 | 91.89 | 93.79 | 2.94 | 0.9 | 9.85 | 12.77 |
| *H. dancoi* B3 (2018) | 36396 | 20807 | 20672.5 | 19434 | 46.44 | 68.5 | 75.21 | 74.92 | 2.74 | 0.87 | 8.11 | 9.67 |
| *H. scotti* C1 (2018) | 58786.33 | 48779 | 48360 | 44694.67 | 77.06 | 242 | 260.58 | 254.96 | 3.03 | 0.87 | 8.15 | 34.1 |
| *H. scotti* C2 (2018) | 42993 | 30738 | 30467 | 28877 | 67.17 | 197 | 207.46 | 206.64 | 3.28 | 0.89 | 9.32 | 28.45 |
| *H. scotti* C3 (2018) | 60614 | 54219 | 53915 | 51533 | 85.02 | 269 | 271.5 | 270.29 | 3.41 | 0.9 | 10.25 | 37.18 |
| SED18a | 106108.3 | 94117.3 | 91107.67 | 64600 | 61.45 | 1223.67 | 1287.31 | 1291.74 | 5.51 | 0.99 | 83.37 | 214.51 |
| WAT18a | 96756 | 85992 | 85010 | 68091 | 70.37 | 394 | 516.16 | 533.18 | 3.89 | 0.96 | 22.28 | 55.37 |
| SED19a | 47261 | 41407 | 40034 | 31525 | 66.70 | 1093 | 1171.65 | 1176.1 | 5.37 | 0.99 | 78.48 | 219.80 |
| SED19b | 124044 | 109284.5 | 106152.5 | 81051.5 | 63.99 | 1527.5 | 1622.15 | 1619.44 | 5.47 | 0.99 | 77.89 | 272.44 |
| WAT19a | 77612 | 67050 | 65846 | 56005 | 72.16 | 591 | 655.28 | 648.7 | 3.54 | 0.91 | 10.60 | 92.18 |
| WAT19b | 88212 | 78483.5 | 77243 | 62434.5 | 70.72 | 606 | 670.54 | 665.48 | 3.85 | 0.93 | 15.75 | 93.72 |

**Supplementary Table S4.** Main data on ASV relative abundance (%) at order level for samples included in this study.

| **Order** | ***H.dancoi* B1** | ***H.dancoi* B3** | ***H. dancoi* 1Sp2a** | ***H.scotti* C1** | ***H.scotti* C2** | ***H.scotti* C3** | ***H. scotti* 1Sp1a** | ***H. scotti* 1Sp1b** | ***H. scotti* 1Sp1c** | **SED18a** | **SED19a** | **SED19b** | **WAT18a** | **WAT19a** | **WAT19b** |
| --- | --- | --- | --- | --- | --- | --- | --- | --- | --- | --- | --- | --- | --- | --- | --- |
| *Actinomarinales* | 0.0 | 0.0 | 0.0 | 0.0 | 0.0 | 0.0 | 0.0 | 0.0 | 0.0 | 1.9 | 3.4 | 3.2 | 0.0 | 0.0 | 0.0 |
| *Aminicenantales* | 0.0 | 0.0 | 0.0 | 0.0 | 0.0 | 0.0 | 0.0 | 0.0 | 0.0 | 0.1 | 0.2 | 0.3 | 0.0 | 0.0 | 0.0 |
| *Anaerolineales* | 0.0 | 0.0 | 0.0 | 0.0 | 0.0 | 0.0 | 0.0 | 0.0 | 0.0 | 0.9 | 2.0 | 2.0 | 0.0 | 0.0 | 0.0 |
| *Arenicellales* | 0.0 | 0.0 | 0.0 | 0.0 | 0.0 | 0.0 | 0.0 | 0.0 | 0.0 | 0.3 | 0.0 | 0.0 | 0.0 | 0.0 | 0.2 |
| AT-s2-59 | 0.0 | 0.0 | 0.0 | 0.0 | 0.0 | 0.0 | 0.0 | 0.0 | 0.0 | 0.1 | 0.0 | 0.0 | 0.0 | 0.0 | 0.0 |
| B2M28 | 0.0 | 0.0 | 0.0 | 0.0 | 0.0 | 0.0 | 0.0 | 0.0 | 0.0 | 0.1 | 0.3 | 0.3 | 0.0 | 0.0 | 0.0 |
| *Babeliales* | 0.0 | 0.0 | 0.3 | 0.0 | 0.0 | 0.0 | 0.0 | 0.0 | 0.0 | 0.0 | 0.1 | 0.2 | 0.0 | 0.0 | 0.0 |
| *Bacillales* | 1.7 | 4.5 | 5.3 | 0.0 | 0.5 | 0.3 | 0.2 | 0.1 | 0.0 | 0.0 | 0.1 | 0.1 | 0.0 | 0.3 | 0.1 |
| *Bacteroidales* | 11.8 | 6.8 | 0.0 | 0.0 | 0.0 | 0.0 | 0.0 | 0.0 | 0.0 | 2.4 | 1.2 | 1.1 | 0.0 | 0.0 | 0.1 |
| *Bacteroidetes VC2.1 Bac22* | 0.0 | 0.0 | 0.0 | 0.0 | 0.0 | 0.0 | 0.0 | 0.0 | 0.0 | 0.3 | 0.1 | 0.1 | 0.0 | 0.0 | 0.0 |
| BD7-8 | 0.0 | 0.0 | 0.0 | 0.0 | 0.0 | 0.0 | 0.0 | 0.0 | 0.0 | 0.4 | 0.0 | 0.0 | 0.0 | 0.0 | 0.0 |
| *Bdellovibrionales* | 0.0 | 0.0 | 0.0 | 0.5 | 0.0 | 0.1 | 0.5 | 0.3 | 2.2 | 0.0 | 0.0 | 0.0 | 0.0 | 0.0 | 0.0 |
| *Bifidobacteriales* | 1.4 | 0.0 | 0.0 | 0.0 | 0.0 | 0.0 | 0.0 | 0.0 | 0.0 | 0.0 | 0.0 | 0.0 | 0.0 | 0.0 | 0.0 |
| *Burkholderiales* | 3.8 | 5.8 | 3.3 | 0.1 | 0.3 | 0.2 | 0.5 | 0.0 | 0.3 | 0.1 | 0.1 | 0.0 | 0.5 | 0.3 | 0.5 |
| *Caldilineales* | 0.0 | 0.0 | 0.0 | 0.0 | 0.0 | 0.0 | 0.0 | 0.0 | 0.0 | 0.2 | 0.0 | 0.1 | 0.0 | 0.0 | 0.0 |
| *Campylobacterales* | 0.0 | 0.0 | 0.5 | 0.0 | 0.0 | 0.0 | 0.0 | 0.0 | 0.0 | 0.0 | 10.6 | 11.5 | 0.0 | 0.0 | 0.6 |
| Candidatus Kerfeldbacteria | 0.0 | 0.0 | 0.0 | 0.0 | 0.0 | 0.0 | 0.0 | 0.0 | 0.0 | 0.0 | 0.0 | 0.1 | 0.0 | 0.0 | 0.0 |
| Candidatus Moranbacteria | 0.0 | 0.0 | 0.0 | 0.0 | 0.0 | 0.0 | 0.0 | 0.0 | 0.0 | 0.1 | 0.5 | 0.6 | 0.0 | 0.1 | 0.0 |
| Candidatus *Peregrinibacteria* | 0.0 | 0.0 | 0.0 | 0.0 | 0.0 | 0.0 | 0.0 | 0.0 | 0.0 | 0.0 | 0.2 | 0.2 | 0.0 | 0.0 | 0.0 |
| Candidatus Uhrbacteria | 0.0 | 0.0 | 0.0 | 0.0 | 0.0 | 0.0 | 0.0 | 0.0 | 0.0 | 0.0 | 0.1 | 0.1 | 0.0 | 0.0 | 0.0 |
| *Caulobacterales* | 0.0 | 0.0 | 0.0 | 0.0 | 0.0 | 0.0 | 0.0 | 0.0 | 0.0 | 0.0 | 0.0 | 0.0 | 0.0 | 0.0 | 0.2 |
| CCM11a | 0.0 | 0.0 | 0.0 | 0.0 | 0.0 | 0.0 | 0.2 | 0.0 | 0.0 | 0.0 | 0.0 | 0.0 | 0.0 | 0.0 | 0.0 |
| *Chitinophagales* | 0.0 | 0.0 | 0.0 | 0.1 | 0.0 | 0.0 | 0.1 | 0.0 | 0.0 | 0.2 | 0.1 | 0.1 | 0.5 | 0.1 | 1.1 |
| *Chlamydiales* | 0.0 | 0.0 | 0.5 | 0.1 | 0.0 | 0.4 | 0.1 | 0.0 | 0.0 | 0.1 | 0.2 | 0.2 | 0.0 | 0.3 | 0.0 |
| *Christensenellales* | 0.0 | 0.0 | 0.0 | 0.0 | 0.0 | 0.0 | 0.0 | 0.0 | 0.0 | 0.1 | 0.5 | 0.5 | 0.0 | 0.0 | 0.0 |
| *Chromatiales* | 0.0 | 0.0 | 0.0 | 0.0 | 0.0 | 0.0 | 0.0 | 0.0 | 0.0 | 0.2 | 0.0 | 0.0 | 0.0 | 0.0 | 0.0 |
| *Cloacimonadales* | 0.0 | 0.0 | 0.0 | 0.0 | 0.0 | 0.0 | 0.0 | 0.0 | 0.0 | 0.2 | 0.2 | 0.1 | 0.0 | 0.0 | 0.0 |
| *Clostridia UCG-014* | 0.0 | 0.0 | 0.0 | 0.0 | 0.0 | 0.0 | 0.0 | 0.0 | 0.0 | 0.1 | 0.0 | 0.0 | 0.0 | 0.0 | 0.0 |
| Order | *H.dancoi* B1 | *H.dancoi* B3 | *H. dancoi* 1Sp2a | *H.scotti* C1 | *H.scotti* C2 | *H.scotti* C3 | *H. scotti* 1Sp1a | *H. scotti* 1Sp1b | *H. scotti* 1Sp1c | SED18a | SED19a | SED19b | WAT18a | WAT19a | WAT19b |
| *Clostridiales* | 0.0 | 0.0 | 0.0 | 0.0 | 0.0 | 0.0 | 0.0 | 0.0 | 0.0 | 0.1 | 0.4 | 0.4 | 0.0 | 0.0 | 0.0 |
| *Coriobacteriales* | 0.0 | 0.0 | 0.0 | 0.0 | 0.0 | 0.0 | 0.0 | 0.0 | 0.0 | 0.2 | 0.0 | 0.0 | 0.0 | 0.0 | 0.0 |
| *Corynebacteriales* | 0.4 | 0.7 | 0.0 | 0.0 | 0.6 | 0.0 | 0.0 | 0.0 | 0.0 | 0.0 | 0.0 | 0.0 | 0.0 | 0.0 | 0.0 |
| *Coxiellales* | 0.0 | 0.0 | 0.0 | 0.0 | 0.0 | 0.0 | 0.1 | 0.0 | 0.0 | 0.0 | 0.0 | 0.0 | 0.0 | 0.0 | 0.0 |
| *Cytophagales* | 0.0 | 0.0 | 0.0 | 0.0 | 0.0 | 0.0 | 0.2 | 0.0 | 0.0 | 0.4 | 0.1 | 0.1 | 0.1 | 0.0 | 0.0 |
| *Defluviicoccales* | 0.0 | 0.0 | 0.0 | 0.0 | 0.0 | 0.0 | 0.0 | 0.0 | 0.0 | 0.1 | 0.0 | 0.0 | 0.0 | 0.0 | 0.0 |
| *Desulfatiglandales* | 0.0 | 0.0 | 0.0 | 0.0 | 0.0 | 0.0 | 0.0 | 0.0 | 0.0 | 0.2 | 0.1 | 0.1 | 0.0 | 0.0 | 0.0 |
| *Desulfitibacterales* | 2.9 | 0.0 | 0.0 | 0.0 | 1.4 | 0.0 | 0.0 | 0.0 | 0.0 | 0.0 | 0.0 | 0.0 | 0.0 | 0.0 | 0.0 |
| *Desulfitobacteriales* | 0.0 | 0.0 | 0.0 | 0.0 | 0.0 | 0.0 | 0.0 | 0.0 | 0.0 | 0.0 | 0.1 | 0.0 | 0.0 | 0.0 | 0.0 |
| *Desulfobacterales* | 0.0 | 0.0 | 0.0 | 0.0 | 0.0 | 0.0 | 0.0 | 0.0 | 0.0 | 3.7 | 1.6 | 1.5 | 0.0 | 0.0 | 0.0 |
| *Desulfobulbales* | 0.0 | 0.0 | 0.0 | 0.0 | 0.0 | 0.0 | 0.0 | 0.0 | 0.0 | 5.2 | 5.3 | 5.1 | 0.0 | 0.0 | 0.0 |
| *Desulfuromonadales* | 0.0 | 0.0 | 0.0 | 0.0 | 0.0 | 0.0 | 0.0 | 0.0 | 0.0 | 0.0 | 0.1 | 0.0 | 0.0 | 0.0 | 0.0 |
| *Ectothiorhodospirales* | 0.0 | 0.0 | 0.0 | 0.0 | 0.1 | 0.0 | 0.1 | 0.0 | 0.0 | 0.0 | 0.0 | 0.0 | 0.0 | 0.0 | 0.0 |
| *Enterobacterales* | 12.1 | 12.5 | 13.3 | 0.2 | 1.2 | 0.1 | 0.5 | 0.8 | 0.2 | 0.0 | 0.0 | 0.1 | 1.8 | 0.2 | 3.3 |
| *Erysipelotrichales* | 0.0 | 2.1 | 2.6 | 0.2 | 0.3 | 0.4 | 0.0 | 0.3 | 0.0 | 0.1 | 0.0 | 0.0 | 0.0 | 0.0 | 0.0 |
| *Flavobacteriales* | 2.7 | 2.7 | 10.5 | 25.4 | 25.6 | 26.9 | 6.0 | 0.5 | 12.2 | 27.1 | 17.5 | 17.1 | 38.9 | 21.0 | 23.2 |
| *Frankiales* | 0.0 | 0.6 | 0.0 | 0.0 | 0.0 | 0.0 | 0.0 | 0.0 | 0.0 | 0.0 | 0.0 | 0.0 | 0.0 | 0.0 | 0.0 |
| *Fusobacteriales* | 0.0 | 0.0 | 0.0 | 0.0 | 0.0 | 0.0 | 0.0 | 0.0 | 0.0 | 0.3 | 0.0 | 0.0 | 0.0 | 0.0 | 0.2 |
| *Gammaproteobacteria Incertae Sedis* | 0.0 | 0.0 | 0.0 | 0.0 | 0.0 | 0.0 | 0.3 | 0.0 | 0.0 | 2.9 | 1.1 | 1.1 | 0.0 | 0.0 | 0.0 |
| *Granulosicoccales* | 0.0 | 0.0 | 0.0 | 0.0 | 0.0 | 0.0 | 0.0 | 0.0 | 0.0 | 0.0 | 0.1 | 0.1 | 0.0 | 0.0 | 0.1 |
| HglApr721 | 0.0 | 0.0 | 0.3 | 0.0 | 0.0 | 0.0 | 0.0 | 0.0 | 0.0 | 0.0 | 0.0 | 0.0 | 0.0 | 0.0 | 0.0 |
| HOC36 | 0.0 | 0.0 | 0.0 | 0.0 | 0.0 | 0.0 | 0.0 | 0.0 | 0.0 | 1.5 | 0.0 | 0.0 | 0.0 | 0.0 | 0.0 |
| *Ignavibacteriales* | 0.0 | 0.0 | 0.0 | 0.0 | 0.0 | 0.0 | 0.0 | 0.0 | 0.0 | 0.1 | 0.1 | 0.1 | 0.0 | 0.0 | 0.0 |
| JGI 0000069-P22 | 0.0 | 0.0 | 0.0 | 0.0 | 0.0 | 0.0 | 0.0 | 0.0 | 0.0 | 0.0 | 0.0 | 0.0 | 0.0 | 0.1 | 0.0 |
| JTB23 | 0.0 | 0.0 | 0.0 | 0.0 | 0.0 | 0.0 | 0.1 | 0.0 | 0.0 | 0.0 | 0.0 | 0.0 | 0.0 | 0.0 | 0.0 |
| *Kiritimatiellales* | 0.0 | 0.0 | 0.0 | 0.0 | 0.0 | 0.0 | 0.0 | 0.0 | 0.0 | 0.1 | 0.0 | 0.0 | 0.0 | 0.0 | 0.0 |
| *Lachnospirales* | 1.0 | 4.1 | 0.0 | 0.1 | 0.3 | 0.0 | 0.0 | 0.0 | 0.0 | 0.1 | 1.1 | 1.0 | 0.0 | 0.0 | 0.1 |
| *Lactobacillales* | 5.2 | 3.6 | 0.0 | 0.0 | 0.2 | 0.0 | 0.0 | 0.2 | 0.0 | 0.0 | 0.1 | 0.1 | 0.0 | 0.0 | 0.0 |
| *Latescibacterales* | 0.0 | 0.0 | 0.0 | 0.0 | 0.0 | 0.0 | 0.0 | 0.0 | 0.0 | 0.2 | 0.1 | 0.1 | 0.0 | 0.0 | 0.0 |
| LD1-PB3 | 0.0 | 0.0 | 0.0 | 0.0 | 0.0 | 0.0 | 0.0 | 0.0 | 0.0 | 0.1 | 0.0 | 0.1 | 0.0 | 0.0 | 0.0 |
| *Legionellales* | 0.0 | 0.0 | 0.0 | 0.0 | 0.0 | 0.0 | 0.0 | 0.0 | 0.0 | 0.0 | 0.1 | 0.1 | 0.0 | 2.2 | 0.0 |
| Order | *H.dancoi* B1 | *H.dancoi* B3 | *H. dancoi* 1Sp2a | *H.scotti* C1 | *H.scotti* C2 | *H.scotti* C3 | *H. scotti* 1Sp1a | *H. scotti* 1Sp1b | *H. scotti* 1Sp1c | SED18a | SED19a | SED19b | WAT18a | WAT19a | WAT19b |
| *Micavibrionales* | 0.0 | 0.0 | 0.0 | 0.0 | 0.0 | 0.0 | 0.1 | 0.0 | 0.0 | 0.0 | 0.0 | 0.1 | 0.0 | 0.1 | 0.0 |
| *Micrococcales* | 0.6 | 0.1 | 0.0 | 0.0 | 0.2 | 0.0 | 0.0 | 0.0 | 0.0 | 0.0 | 0.0 | 0.0 | 0.0 | 0.0 | 0.0 |
| *Microtrichales* | 0.0 | 0.0 | 0.0 | 0.0 | 0.0 | 0.0 | 0.2 | 0.0 | 0.0 | 3.9 | 2.5 | 2.3 | 0.1 | 0.2 | 0.2 |
| *Myxococcales* | 0.0 | 0.0 | 0.0 | 0.0 | 0.0 | 0.0 | 0.0 | 0.0 | 0.0 | 0.0 | 0.1 | 0.1 | 0.0 | 0.0 | 0.0 |
| *Nitrosococcales* | 0.8 | 0.0 | 2.4 | 0.5 | 0.2 | 1.9 | 0.4 | 0.0 | 0.1 | 0.0 | 0.0 | 0.0 | 2.3 | 0.5 | 1.6 |
| *Nitrospinales* | 0.0 | 0.0 | 0.0 | 4.0 | 1.2 | 0.6 | 1.0 | 0.4 | 2.2 | 0.0 | 0.0 | 0.0 | 0.0 | 0.0 | 0.0 |
| OPB41 | 0.0 | 0.0 | 0.0 | 0.0 | 0.0 | 0.0 | 0.0 | 0.0 | 0.0 | 0.0 | 0.1 | 0.1 | 0.0 | 0.0 | 0.0 |
| *Opitutales* | 0.0 | 0.0 | 0.0 | 0.0 | 0.0 | 0.0 | 0.0 | 0.0 | 0.0 | 0.5 | 0.1 | 0.1 | 0.1 | 0.0 | 0.0 |
| *Oscillospirales* | 0.5 | 0.9 | 0.0 | 0.0 | 0.0 | 0.3 | 0.0 | 0.0 | 0.0 | 0.8 | 0.6 | 0.6 | 0.0 | 0.0 | 0.0 |
| *Parvibaculales* | 0.0 | 0.0 | 0.0 | 0.1 | 0.0 | 0.0 | 0.3 | 0.0 | 0.0 | 0.0 | 0.1 | 0.1 | 0.0 | 0.1 | 0.0 |
| PB19 | 0.0 | 0.0 | 0.0 | 0.0 | 0.2 | 0.0 | 0.0 | 0.0 | 0.0 | 0.0 | 0.0 | 0.0 | 0.0 | 0.0 | 0.0 |
| PeM15 | 0.0 | 0.0 | 0.7 | 0.0 | 0.0 | 0.0 | 0.0 | 0.0 | 0.0 | 0.1 | 0.0 | 0.0 | 0.0 | 0.1 | 0.0 |
| *Peptococcales* | 0.0 | 0.0 | 0.0 | 0.0 | 0.0 | 0.0 | 0.0 | 0.0 | 0.0 | 0.0 | 0.1 | 0.1 | 0.0 | 0.0 | 0.0 |
| *Peptostreptococcales-Tissierellales* | 0.5 | 0.0 | 0.0 | 0.1 | 0.0 | 0.8 | 0.0 | 0.0 | 0.0 | 0.7 | 2.6 | 2.5 | 0.0 | 0.0 | 0.0 |
| *Phycisphaerales* | 0.0 | 0.0 | 1.0 | 0.0 | 0.0 | 1.0 | 0.1 | 0.0 | 0.6 | 0.3 | 0.1 | 0.1 | 0.0 | 0.0 | 0.0 |
| *Pirellulales* | 0.0 | 0.2 | 5.3 | 0.0 | 0.0 | 0.0 | 0.7 | 0.1 | 0.0 | 7.4 | 4.9 | 5.3 | 0.0 | 0.2 | 0.1 |
| *pItb-vmat-80* | 0.0 | 0.0 | 0.0 | 0.0 | 0.0 | 0.0 | 0.1 | 0.0 | 0.0 | 0.0 | 0.0 | 0.0 | 0.0 | 0.0 | 0.0 |
| *Planctomycetales* | 0.0 | 0.0 | 0.0 | 0.0 | 0.0 | 0.0 | 0.3 | 0.0 | 0.0 | 0.3 | 0.5 | 0.5 | 0.0 | 0.2 | 0.0 |
| *Polyangiales* | 0.0 | 0.0 | 0.0 | 0.0 | 0.0 | 0.0 | 0.0 | 0.0 | 0.0 | 1.5 | 0.1 | 0.1 | 0.0 | 0.0 | 0.0 |
| *Propionibacteriales* | 38.0 | 39.1 | 2.0 | 0.9 | 6.5 | 2.2 | 0.1 | 0.4 | 0.1 | 0.0 | 0.1 | 0.1 | 0.0 | 0.0 | 0.1 |
| *Pseudomonadales* | 7.1 | 7.9 | 3.2 | 17.3 | 13.8 | 16.7 | 41.9 | 37.4 | 43.1 | 2.0 | 8.3 | 8.1 | 29.2 | 27.6 | 14.8 |
| *Puniceispirillales* | 0.0 | 0.0 | 0.0 | 0.0 | 0.0 | 0.0 | 0.0 | 0.0 | 0.0 | 0.0 | 0.0 | 0.0 | 0.2 | 0.0 | 0.1 |
| *Rhizobiales* | 0.3 | 1.2 | 0.0 | 0.0 | 0.0 | 0.3 | 0.6 | 0.0 | 0.0 | 0.5 | 0.5 | 0.4 | 0.0 | 0.5 | 0.1 |
| *Rhodobacterales* | 0.2 | 0.7 | 6.9 | 1.9 | 1.0 | 0.8 | 2.9 | 0.1 | 0.2 | 1.6 | 2.5 | 2.8 | 14.5 | 33.3 | 36.6 |
| *Rhodospirillales* | 0.0 | 0.0 | 0.7 | 0.0 | 0.0 | 0.1 | 0.0 | 0.0 | 0.0 | 0.0 | 0.0 | 0.0 | 0.2 | 0.0 | 0.0 |
| *Rickettsiales* | 0.3 | 0.0 | 17.7 | 0.6 | 2.2 | 5.0 | 0.3 | 0.5 | 0.0 | 0.0 | 0.0 | 0.1 | 0.0 | 0.0 | 0.0 |
| *Saccharimonadales* | 0.0 | 0.0 | 0.6 | 0.0 | 0.0 | 0.0 | 0.2 | 0.0 | 0.0 | 0.3 | 0.3 | 0.4 | 0.0 | 0.1 | 0.0 |
| SAR11 clade | 0.0 | 0.0 | 0.0 | 2.1 | 4.0 | 0.5 | 0.0 | 0.0 | 0.3 | 0.0 | 0.0 | 0.0 | 3.9 | 3.6 | 3.0 |
| SBR1031 | 0.0 | 0.0 | 0.0 | 0.0 | 0.0 | 0.0 | 0.0 | 0.0 | 0.0 | 0.0 | 0.0 | 0.1 | 0.0 | 0.0 | 0.0 |
| SJA-15 | 0.0 | 0.0 | 0.0 | 0.0 | 0.0 | 0.0 | 0.0 | 0.0 | 0.0 | 0.1 | 0.1 | 0.1 | 0.0 | 0.0 | 0.0 |
| *Sneathiellales* | 0.0 | 0.0 | 0.0 | 0.0 | 0.0 | 0.0 | 0.2 | 0.0 | 0.0 | 0.0 | 0.0 | 0.0 | 0.0 | 0.0 | 0.0 |
| Order | *H.dancoi* B1 | *H.dancoi* B3 | *H. dancoi* 1Sp2a | *H.scotti* C1 | *H.scotti* C2 | *H.scotti* C3 | *H. scotti* 1Sp1a | *H. scotti* 1Sp1b | *H. scotti* 1Sp1c | SED18a | SED19a | SED19b | WAT18a | WAT19a | WAT19b |
| *Solirubrobacterales* | 0.0 | 0.0 | 0.0 | 0.0 | 0.0 | 0.0 | 0.2 | 0.0 | 0.0 | 0.2 | 0.0 | 0.0 | 0.0 | 0.0 | 0.0 |
| *Sphingobacteriales* | 0.0 | 0.0 | 0.0 | 0.4 | 0.0 | 0.6 | 0.0 | 0.0 | 0.0 | 0.3 | 0.0 | 0.1 | 1.9 | 0.0 | 2.4 |
| *Sphingomonadales* | 0.0 | 1.6 | 0.0 | 0.0 | 0.0 | 0.0 | 1.2 | 0.4 | 0.0 | 0.0 | 0.1 | 0.0 | 0.0 | 0.3 | 0.3 |
| *Spirochaetales* | 0.0 | 0.0 | 0.0 | 0.3 | 0.4 | 0.4 | 0.0 | 0.2 | 0.2 | 0.6 | 0.2 | 0.1 | 0.0 | 0.0 | 0.0 |
| *Staphylococcales* | 3.8 | 1.6 | 0.0 | 0.1 | 0.1 | 0.0 | 0.1 | 0.1 | 0.0 | 0.0 | 0.0 | 0.0 | 0.0 | 0.0 | 0.0 |
| *Steroidobacterales* | 0.0 | 0.0 | 0.0 | 0.0 | 0.0 | 0.0 | 1.9 | 0.0 | 0.0 | 3.3 | 1.1 | 0.9 | 0.0 | 0.0 | 0.1 |
| *Subgroup 17* | 0.0 | 0.0 | 0.0 | 0.0 | 0.0 | 0.0 | 0.2 | 0.0 | 0.0 | 0.1 | 0.1 | 0.1 | 0.0 | 0.0 | 0.0 |
| *Synergistales* | 0.0 | 0.0 | 0.0 | 0.2 | 0.0 | 0.0 | 0.0 | 0.0 | 0.0 | 0.0 | 0.0 | 0.0 | 0.0 | 0.0 | 0.0 |
| *Thermoanaerobaculales* | 0.0 | 0.0 | 0.0 | 0.0 | 0.0 | 0.0 | 0.2 | 0.0 | 0.0 | 4.0 | 1.5 | 1.3 | 0.0 | 0.0 | 0.0 |
| *Thiohalorhabdales* | 0.0 | 0.0 | 0.0 | 0.0 | 0.0 | 0.0 | 0.2 | 0.0 | 0.0 | 0.1 | 0.1 | 0.1 | 0.0 | 0.0 | 0.0 |
| *Thiotrichales* | 0.0 | 0.0 | 0.0 | 0.0 | 0.0 | 0.0 | 0.6 | 0.0 | 0.1 | 0.5 | 5.0 | 4.3 | 0.0 | 0.4 | 0.2 |
| UBA10353 marine group | 0.0 | 0.0 | 0.0 | 39.6 | 34.1 | 32.3 | 28.9 | 54.9 | 34.6 | 0.1 | 0.1 | 0.1 | 0.0 | 0.0 | 0.0 |
| *Verrucomicrobiales* | 0.0 | 0.0 | 0.4 | 0.2 | 0.0 | 0.0 | 4.8 | 0.0 | 0.3 | 11.5 | 13.3 | 13.2 | 0.1 | 7.0 | 2.0 |
| WCHB1-41 | 0.0 | 0.0 | 0.0 | 0.0 | 0.0 | 0.0 | 0.0 | 0.0 | 0.0 | 0.6 | 0.1 | 0.2 | 0.0 | 0.0 | 0.0 |
| NA | 4.7 | 3.1 | 22.2 | 4.4 | 5.4 | 7.9 | 3.6 | 3.3 | 2.7 | 5.9 | 3.7 | 4.0 | 0.1 | 0.2 | 0.2 |
